# Ischemic stroke and sarcopenia have an asymmetric bidirectional relationship based on a two-sample Mendelian randomization study

**DOI:** 10.3389/fneur.2024.1427692

**Published:** 2024-10-09

**Authors:** Fan-Qiao Meng, Yu Zhang, Xiao-Xin Bai, Fan-Li Kong, Feng-E Li

**Affiliations:** ^1^Department of Postgraduate, School of Clinical Medicine, Beihua University, Jilin, China; ^2^Department of Pediatrics, The First Hospital of Jilin University, Changchun, China; ^3^Department of Pathophysiology, School of Basic Medicine, Beihua University, Jilin, China; ^4^Department of Neurology, Second Affiliated Hospital of Shantou University Medical College, Shantou, Guangdong, China; ^5^Department of Neurology, The Affiliated Hospital of Beihua University, Jilin, China

**Keywords:** ischemic stroke, sarcopenia, Mendelian randomization, causality, genetics

## Abstract

**Background:**

We investigated the potential relationship between age-related conditions, particularly sarcopenia and ischemic stroke (IS), through a two-sample Mendelian randomization (MR) study.

**Methods:**

We conducted a two-sample bidirectional MR study to investigate the relationship between sarcopenia and stroke. Genetic instruments for sarcopenia were derived from the UK Biobank, while data on IS and its subtypes were obtained from the MEGASTROKE consortium. Inverse variance weighting (IVW) served as the primary analytical method. Additionally, heterogeneity and pleiotropy were assessed to ensure the robustness of the findings.

**Results:**

The analysis indicates a negative correlation between appendicular lean mass (ALM) and small vessel stroke (SVS; OR = 0.790, 95% CI: 0.703–0.888, *p* < 0.001), a positive correlation with cardioembolic stroke (CES; OR = 1.165, 95% CI: 1.058–1.284, *p* = 0.002), and no causal relationship with any ischemic stroke (AIS) or large artery stroke (LAS). Additionally, SVS is negatively associated with right-hand grip strength (OR = 0.639, 95% CI: 0.437–0.934, *p* = 0.021), while AIS, LAS, and CES do not exhibit a causal relationship with grip strength. Furthermore, no causal relationship was identified between left-hand grip strength, usual walking pace, and IS or its subtypes. MR analysis reveals only a negative association between CES and usual walking pace (OR = 0.989, 95% CI: 0.980–0.998, *p* = 0.013), with no associations found between other IS subtypes and sarcopenia-related traits.

**Conclusion:**

This study demonstrates that a reduction in ALM and right-hand grip strength is associated with SVS, whereas decreased ALM may serve as a protective factor against CES. Conversely, our analysis suggests that CES can impact walking speed. Overall, these findings provide valuable insights into the prevention and treatment of these conditions.

## 1 Introduction

Stroke is the second leading cause of death and disability globally (1). In China, the incidence of stroke is rising each year, making it the leading cause of mortality (2, 3). According to a 2019 study, common risk factors for stroke include hypertension, high body mass index (BMI), elevated fasting glucose, air pollution, and smoking (4). However, given the increasing incidence of stroke, it is crucial to explore additional risk factors. Sarcopenia, which has been identified as influencing the incidence and outcomes of stroke, has primarily been studied through observational data (5–8). These observational studies are often subject to confounding factors, complicating the establishment of accurate causal relationships. While the impact of sarcopenia on stroke risk has been explored, most studies have focused on the overall risk of stroke without analyzing specific stroke subtypes. Additionally, several studies have reported a relatively high incidence of sarcopenia following a stroke (9–11), which can adversely affect patient recovery and quality of life. Despite this, the relationship between stroke subtypes and sarcopenia has been largely overlooked, with limited research addressing this area. To fill this gap, we employed the Mendelian randomization (MR) method to investigate the causal relationship between sarcopenia and the risk of various stroke subtypes, providing new insights for clinical prevention and intervention strategies.

Sarcopenia was first introduced in the late 1980s by Rosenberg. The term is derived from Greek, with “sarx” meaning flesh and “penia” meaning loss (12). As the global population ages, sarcopenia has emerged as a significant health concern, with an estimated 500 million people projected to be affected by 2050 (13). Sarcopenia is associated with an increased risk of falls, fractures, disability, weakness, and mortality (14). According to the European Working Group on Sarcopenia in Older People (EWGSOP), sarcopenia is defined by a decline in both muscle mass and function (15). In 2018, the EWGSOP2 updated the diagnostic criteria, proposing a stepwise approach. Initially, muscle strength is assessed, typically through grip strength measurement, with reduced grip strength suggesting possible sarcopenia. Next, appendicular lean mass (ALM) is evaluated using dual-energy X-ray absorptiometry, bioelectrical impedance analysis, computed tomography, or magnetic resonance imaging, with a diagnosis confirmed by decreased muscle mass and quantity. Finally, low physical capacity is used to assess the severity of sarcopenia, with commonly used indicators including gait speed and a timed 400-meter walk; reduced performance on these tests indicates severe sarcopenia (16). Consequently, we selected three variables to evaluate the onset and progression of sarcopenia: ALM, grip strength, and walking pace.

MR is a method used to infer causal relationships between an exposure and an outcome, grounded in Mendel’s Second Law. This approach reduces confounding bias and addresses the limitations inherent in traditional observational studies. By selecting single nucleotide polymorphisms (SNPs) associated with the exposure as instrumental variables (IVs), genetic variation is utilized to derive robust causal inferences between exposure factors and outcomes (17). In this study, we employed a two-sample MR analysis to evaluate the causal relationship between sarcopenia and IS.

## 2 Materials and methods

### 2.1 Study design

**Figure 1:**
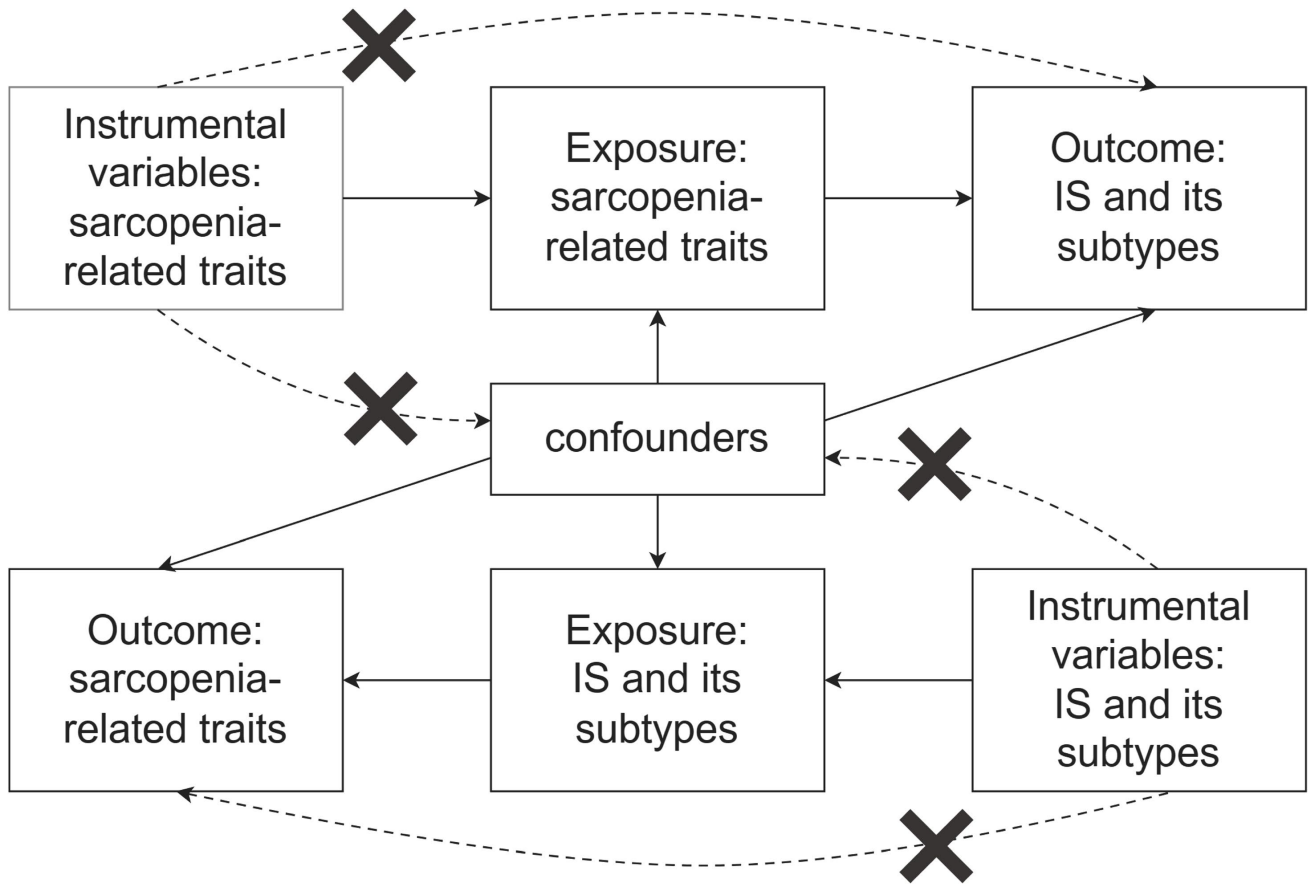
Plot of three assumptions of MR (by Figdraw 2.0).

The MR study was carried out in two distinct phases. Initially, a forward MR analysis was performed, utilizing three key characteristics of sarcopenia as exposures and various stroke subtypes as outcomes. In the second phase, a reverse MR analysis was conducted, where the roles of exposure and outcome were inverted. To ensure the validity of the MR analysis, three key assumptions must be satisfied (Figure 1): (1) The selected genetic instruments must be strongly associated with the exposure, fulfilling the relevance assumption; (2) The chosen genetic instruments must not be related to any confounding factors, adhering to the independence assumption; (3) The genetic instruments should affect the outcome only through the exposure, satisfying the exclusion restriction assumption (18). For this study, we utilized publicly available, large-scale genome-wide association study (GWAS) meta-data sets that had been previously published. In the original GWAS studies, all participants provided informed consent.

### 2.2 Data sources

The EWGSOP2 defines the cut-off values for muscle mass as ALM < 20 kg for men and < 15 kg for women, and ALM/height^2^ < 7.0 kg/m^2^ for men and < 5.5 kg/m^2^ for women. The thresholds for low grip strength are <27 kg for men and < 16 kg for women, while the cut-off for walking pace is ≤0.8 m/s (16). ALM was measured using bioelectrical impedance analysis, based on data from the UK Biobank (n = 450,234) (19). GWAS summary data were also obtained from the UK Biobank for left-hand grip strength (n = 461,089) and right-hand grip strength (n = 461,026) (20). Additionally, the UK Biobank collected data on usual walking pace from 459,915 European individuals through touchscreen questionnaires (20).

The stroke GWAS dataset was sourced from the MEGASTROKE consortium and comprises 40,585 cases and 406,111 controls (21). IS is categorized by etiology into large artery stroke (LAS), small vessel stroke (SVS), and cardioembolic stroke (CES) based on the Trial of Org 10,172 in acute stroke treatment classification. To reduce bias from population stratification, all individuals included in the study were of European descent. Table 1 provides detailed information on the GWAS dataset utilized for the MR analysis.

**Table 1:** Data sources for IS and its subtypes and sarcopenia-related traits.

Type	Sample size	Population	Consortium	GWAS ID/ PubMed ID
ALM	450,243	European	UK Biobank	ebi-a-GCST90000025/33097823
Hand grip strength (left)	461,026	European	UK Biobank	ukb-b-7478/NA
Hand grip strength (right)	461,089	European	UK Biobank	ukb-b-10215/NA
Usual walking pace	459,915	European	UK Biobank	ukb-b-4711/NA
AIS	440,328	European	MEGASTROKE	ebi-a-GCST006908/29531354
LAS	150,765	European	MEGASTROKE	ebi-a-GCST006907/29531354
CES	211,763	European	MEGASTROKE	ebi-a-GCST006910/29531354
SVS	98,048	European	MEGASTROKE	ebi-a-GCST006909/29531354

### 2.3 Genetic tool variable selection

First, SNPs with genome-wide significance (*p* < 5.0 × 10^−8^) were selected, and linkage disequilibrium among them was excluded (*r*^2^ < 0.001, kb = 10,000). This step ensured the identification of independent SNP loci that met the correlation assumption. Second, palindromic SNPs with intermediate allele frequencies were removed (Supplementary Table S8). Subsequently, we utilized PhenoScanner (http://www.phenoscanner.medschl.cam.ac.uk) to systematically search for the selected SNPs and exclude those associated with the outcome and potential confounding factors, including diabetes, hypertension, dyslipidemia, coronary artery disease, BMI, smoking, and alcohol consumption (Supplementary Table S9) (22), in order to satisfy the second and third assumptions. Additionally, SNPs with an F-statistic greater than 10 were considered significantly associated with the exposure. The F-statistic was calculated using the formula: F = [R^2^ / (1-R^2^) × (n-2), R^2^ = 2 × MAF × (1-MAF) × β^2^]. N represents the sample size, and MAF represents minor allele frequency. Weak IVs with *F* values <10 were excluded (23). Finally, MRPRESSO was employed to identify and remove outlier SNPs from the final set of IVs. Proxy SNPs were not included in this MR analysis.

### 2.4 MR analysis

For MR analysis, inverse variance weighting (IVW), weighted median, and MR-Egger were utilized as the primary analytical methods. IVW was employed as the main method, sequentially combining the Wald ratio estimates of each SNP to determine the overall causal effect (24). The weighted median and MR-Egger methods were used as complementary approaches to IVW. When the selected IVs comprise more than half of the total IVs, the weighted median method can reliably provide accurate estimates (25). MR-Egger is capable of detecting and adjusting for horizontal pleiotropy, though it has limited statistical power (26). Therefore, in this study, MR-Egger was applied primarily to test for pleiotropy rather than to evaluate causal effects.

### 2.5 Sensitivity analysis

The robustness of the results and potential biases were evaluated using sensitivity analysis methods in the MR study. Heterogeneity was assessed through Cochran’s Q test and funnel plots (27). To evaluate horizontal pleiotropy, the MR-Egger intercept test was conducted by calculating the intercept via linear regression analysis (28). The Leave-One-Out method was also employed, which involves removing individual SNPs and performing MR analysis to determine their influence on the outcomes (29). Additionally, MR-PRESSO was utilized to identify and exclude potential outliers and detect horizontal pleiotropy, thereby minimizing their impact on the causal effect assessment. If MR-PRESSO identified any outliers, the corresponding SNPs were excluded, and MR analysis was re-conducted. The excluded outliers are listed in Supplementary Table S10 (30).

### 2.6 Enrichment analysis

To explore the biological pathways and mechanisms underlying risk genes associated with sarcopenia-related phenotypes, IS, and its subtypes, we performed enrichment analysis using Gene Ontology (GO) and the Kyoto Encyclopedia of Genes and Genomes (KEGG). GO analysis was used to assess gene enrichment within the categories of cellular components, biological processes, and molecular functions. KEGG analysis was conducted to identify gene enrichment in specific metabolic pathways, which is crucial for understanding genomic interactions.

Statistical analysis for this study was performed using R software (version 4.3.2). The TwoSampleMR (version 0.5.8) and MR-PRESSO (version 1.0) packages were applied for MR analysis, and the clusterProfiler (version 4.4.4) package was used for enrichment analysis.

## 3 Results

Using sarcopenia-related traits as exposures, 617 SNPs for ALM, 140 SNPs for left-hand grip strength, 161 SNPs for right-hand grip strength, and 36 SNPs for usual walking pace were chosen as IVs (Supplementary Tables S1–S4) for MR analysis. For the reverse MR analysis, with IS and its subtypes as exposures, 7 SNPs for AIS, 3 SNPs for LAS, 4 SNPs for CES, and 0 SNPs for SVS were selected as IVs (Supplementary Tables S5–S7).

### 3.1 The causal effect of sarcopenia on IS

**Figure 2:**
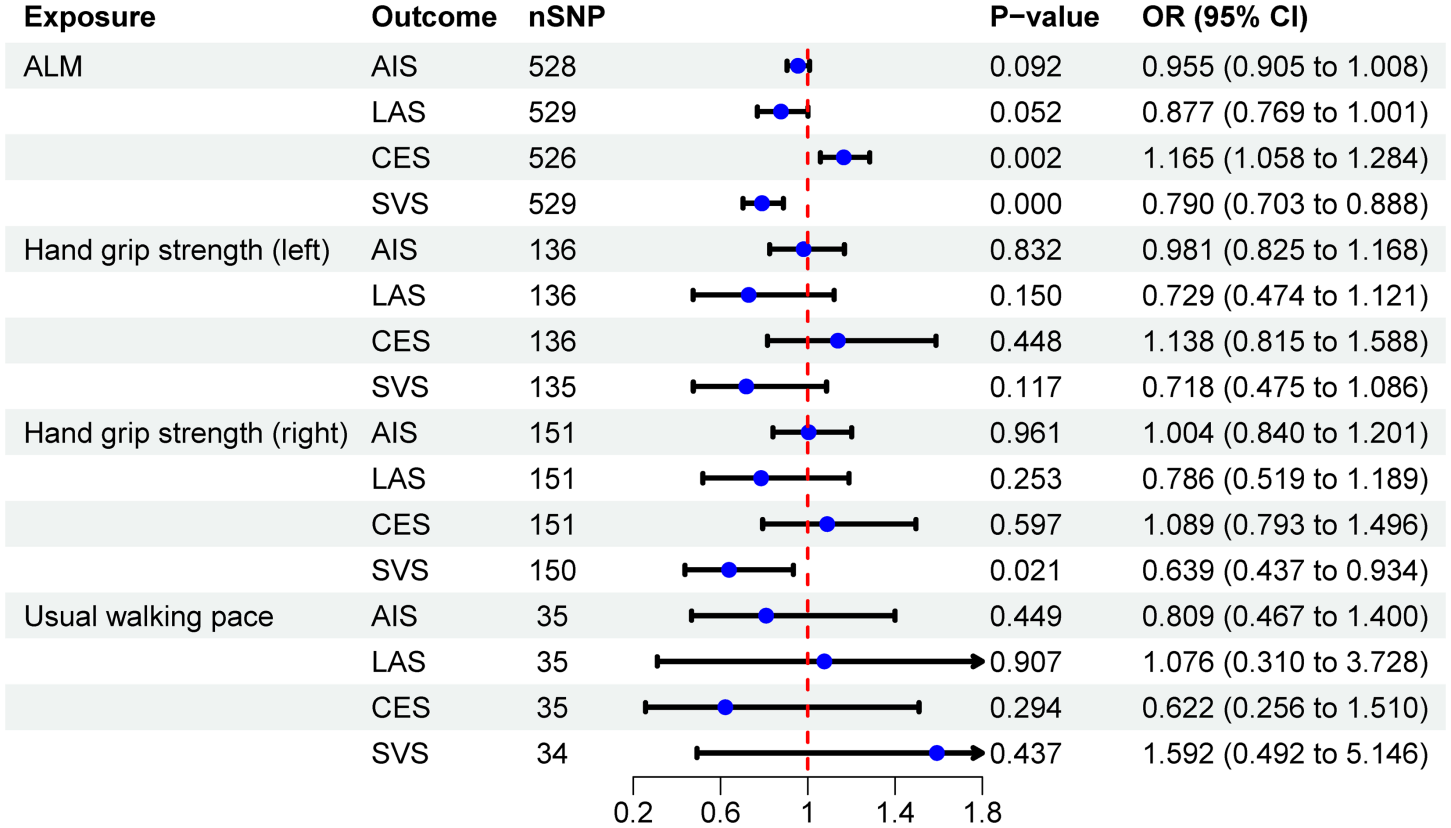
The IVW method estimates the causal effects of sarcopenia on IS.

The IVW analysis revealed a negative correlation between ALM and SVS (OR = 0.790, 95% CI: 0.703–0.888, *p* < 0.001), while a positive correlation was observed between ALM and CES (OR = 1.165, 95% CI: 1.058–1.284, *p* = 0.002). No causal relationship was identified between ALM and either AIS or LAS. Additionally, MR analysis indicated that right-hand grip strength was negatively correlated with SVS (OR = 0.639, 95% CI: 0.437–0.934, *p* = 0.021), with no significant associations with AIS, LAS, or CES. Furthermore, no causal relationships were found between left-hand grip strength, usual walking pace, and IS or its subtypes. The analysis results are detailed in Table 2, with visual representations of the causal relationships provided in Figure 2. Cochran’s Q test, MR-Egger intercept test, and MR-PRESSO global test indicated no evidence of heterogeneity or horizontal pleiotropy (Table 3). The Supplementary Figure include scatter plots from IVW, MR-Egger, and weighted median methods, along with leave-one-out and funnel plots.

**Table 2:** The causal association of sarcopenia on IS by MR analysis results.

Exposure	Outcome	Methods	SNPs	OR (95%CI)	*p*-value
ALM	AIS	IVW	528	0.955 (0.905–1.008)	0.092
		MR Egger	528	0.982 (0.861–1.120)	0.787
		Weighted median	528	1.016 (0.937–1.101)	0.705
ALM	LAS	IVW	529	0.877 (0.769–1.001)	0.052
		MR Egger	529	0.997 (0.720–1.380)	0.984
		Weighted median	529	0.918 (0.761–1.108)	0.373
ALM	CES	IVW	526	1.165 (1.058–1.284)	0.002
		MR Egger	526	1.191 (0.937–1.512)	0.154
		Weighted median	526	1.180 (1.012–1.376)	0.034
ALM	SVS	IVW	529	0.790 (0.703–0.888)	< 0.001
		MR Egger	529	0.686 (0.515–0.915)	0.010
		Weighted median	529	0.786 (0.659–0.938)	0.008
Hand grip strength (left)	AIS	IVW	136	0.981 (0.825–1.168)	0.832
		MR Egger	136	1.590 (0.766–3.301)	0.215
		Weighted median	136	1.064 (0.824–1.373)	0.636
Hand grip strength (left)	LAS	IVW	136	0.729 (0.474–1.121)	0.150
		MR Egger	136	1.524 (0.248–9.379)	0.650
		Weighted median	136	0.716 (0.379–1.351)	0.302
Hand grip strength (left)	CES	IVW	136	1.138 (0.815–1.588)	0.448
		MR Egger	136	1.543 (0.376–6.332)	0.548
		Weighted median	136	1.146 (0.688–1.910)	0.600
Hand grip strength (left)	SVS	IVW	135	0.718 (0.475–1.086)	0.117
		MR Egger	135	0.743 (0.127–4.337)	0.742
		Weighted median	135	0.730 (0.412–1.296)	0.283
Hand grip strength (right)	AIS	IVW	151	1.004 (0.840–1.201)	0.961
		MR Egger	151	1.154 (0.552–2.411)	0.705
		Weighted median	151	1.083 (0.852–1.378)	0.514
Hand grip strength (right)	LAS	IVW	151	0.786 (0.519–1.189)	0.253
		MR Egger	151	0.518 (0.093–2.901)	0.456
		Weighted median	151	0.840 (0.467–1.513)	0.562
Hand grip strength (right)	CES	IVW	151	1.089 (0.793–1.496)	0.597
		MR Egger	151	0.957 (0.255–3.596)	0.949
		Weighted median	151	1.173 (0.746–1.847)	0.490
Hand grip strength (right)	SVS	IVW	150	0.639 (0.437–0.934)	0.021
		MR Egger	150	0.545 (0.111–2.678)	0.456
		Weighted median	150	0.537 (0.307–0.939)	0.029
Usual walking pace	AIS	IVW	35	0.809 (0.467–1.400)	0.449
		MR Egger	35	0.340 (0.022–5.197)	0.444
		Weighted median	35	1.300 (0.649–2.604)	0.460
Usual walking pace	LAS	IVW	35	1.076 (0.310–3.728)	0.907
		MR Egger	35	0.020 (0.000–11.262)	0.235
		Weighted median	35	2.326 (0.471–11.474)	0.300
Usual walking pace	CES	IVW	35	0.622 (0.256–1.510)	0.294
		MR Egger	35	0.289 (0.003–24.804)	0.589
		Weighted median	35	0.288 (0.081–1.025)	0.055
Usual walking pace	SVS	IVW	34	1.592 (0.492–5.146)	0.437
		MR Egger	34	0.751 (0.002–302.402)	0.926
		Weighted median	34	2.139 (0.441–10.368)	0.345

**Table 3:** Sensitivity analysis of sarcopenia to IS.

Exposure	Outcome	IVW		MR Egger				MR-PRESSO *P*-value
		Cochran’s *Q* test	*P*-value	Cochran’s Q test	*P*-value	Intercept	Intercept *P*-value	
ALM	AIS	637.949	6.330E-04	637.699	5.839E-04	-6.555E-04	0.649	7.273E-04
	LAS	630.037	0.001	629.198	0.001	−0.003	0.402	0.002
	CES	556.907	0.162	556.868	0.155	−4.964E-04	0.849	0.128
	SVS	574.353	0.080	573.146	0.080	0.003	0.293	0.086
Hand grip strength (left)	AIS	126.504	0.687	124.725	0.705	−0.006	0.185	0.719
	LAS	130.830	0.585	130.160	0.578	−0.009	0.415	0.584
	CES	126.587	0.685	126.398	0.667	−0.004	0.664	0.713
	SVS	142.481	0.292	142.480	0.271	−3.931E-04	0.969	0.265
Hand grip strength (right)	AIS	179.187	0.052	179.014	0.047	−0.002	0.705	0.027
	LAS	157.622	0.319	157.371	0.303	0.005	0.626	0.260
	CES	153.766	0.400	153.726	0.378	0.002	0.844	0.363
	SVS	151.481	0.428	151.439	0.406	0.002	0.839	0.294
Usual walking pace	AIS	50.513	0.034	49.901	0.030	0.008	0.529	0.014
	LAS	42.064	0.161	40.140	0.183	0.035	0.217	0.158
	CES	35.913	0.379	35.785	0.339	0.007	0.733	0.433
	SVS	40.772	0.166	40.693	0.139	0.007	0.804	0.175

### 3.2 The causal effect of IS on sarcopenia

**Figure 3:**
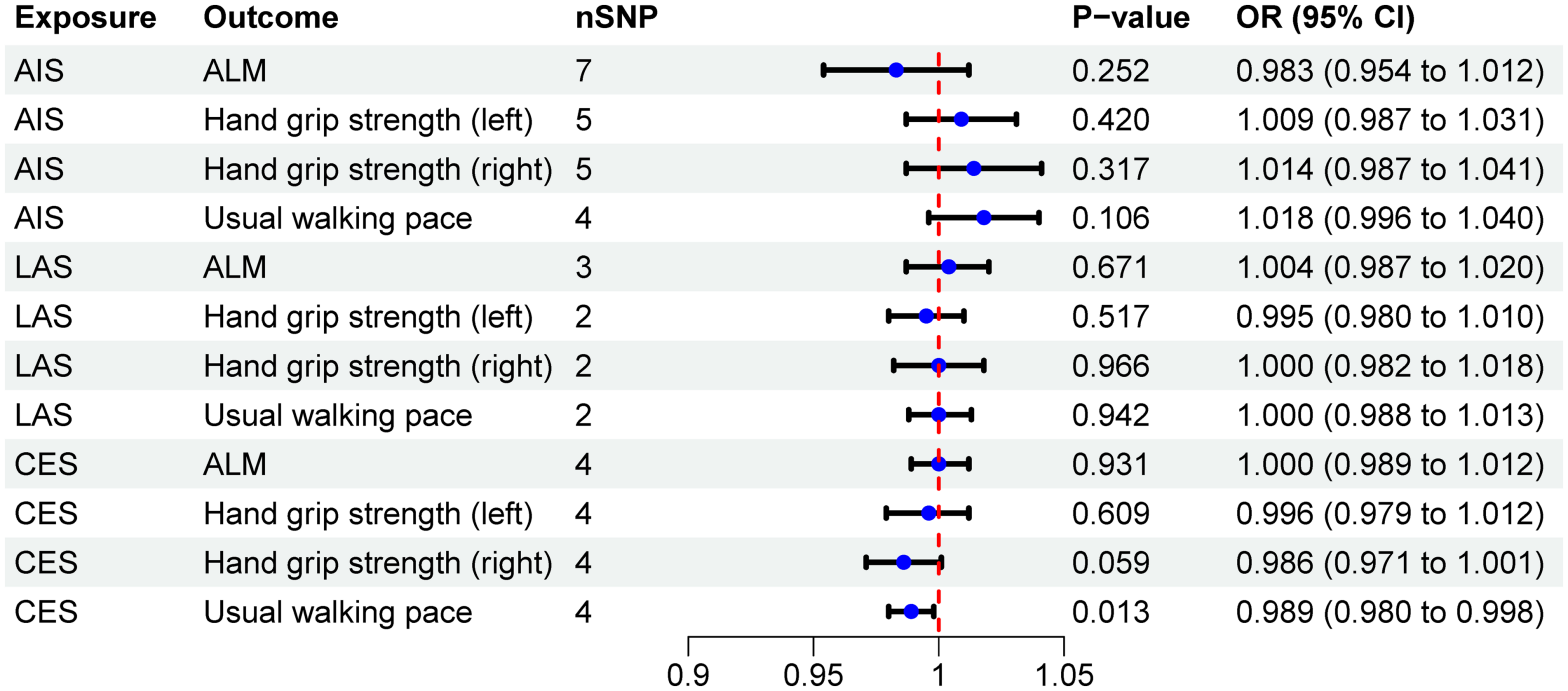
The IVW method estimates the causal effects of IS on sarcopenia.

The MR results demonstrated a negative correlation between CES and usual walking pace (OR = 0.989, 95% CI: 0.980–0.998, *p* = 0.013) (Table 4). However, no significant causal relationships were observed between CES and either ALM or grip strength, as illustrated in Figure 3. Sensitivity analyses, including Cochran’s Q test, MR-Egger intercept test, and MR-PRESSO global test, revealed no evidence of heterogeneity or horizontal pleiotropy (Table 5). Furthermore, the MR analyses did not establish any causal association between AIS, LAS, and sarcopenia-related traits. The Supplementary Figure provide scatter plots from the IVW, MR-Egger, and weighted median methods, along with leave-one-out and funnel plots.

**Table 4:** The causal association of IS on sarcopenia by MR analysis results.

Exposure	Outcome	Methods	SNPs	OR (95%CI)	*P*-value
AIS	ALM	IVW	7	0.983 (0.954–1.012)	0.252
		MR Egger	7	1.110 (0.906–1.359)	0.360
		Weighted median	7	0.983 (0.952–1.016)	0.315
AIS	Hand grip strength (left)	IVW	5	1.009 (0.987–1.031)	0.420
		MR Egger	5	1.015 (0.862–1.196)	0.868
		Weighted median	5	1.003 (0.978–1.028)	0.839
AIS	Hand grip strength (right)	IVW	5	1.014 (0.987–1.041)	0.317
		MR Egger	5	0.921 (0.776–1.092)	0.412
		Weighted median	5	1.011 (0.984–1.039)	0.434
AIS	Usual walking pace	IVW	4	1.018 (0.996–1.040)	0.106
		MR Egger	4	1.006 (0.807–1.255)	0.960
		Weighted median	4	1.016 (0.991–1.042)	0.204
LAS	ALM	IVW	3	1.004 (0.987–1.020)	0.671
		MR Egger	3	0.978 (0.938–1.019)	0.478
		Weighted median	3	1.008 (0.989–1.028)	0.395
LAS	Hand grip strength (left)	IVW	2	0.995 (0.980–1.010)	0.517
		MR Egger	NA	NA	NA
		Weighted median	NA	NA	NA
LAS	Hand grip strength (right)	IVW	2	1.000 (0.982–1.018)	0.966
		MR Egger	NA	NA	NA
		Weighted median	NA	NA	NA
LAS	Usual walking pace	IVW	2	1.000 (0.988–1.013)	0.942
		MR Egger	NA	NA	NA
		Weighted median	NA	NA	NA
CES	ALM	IVW	4	1.000 (0.989–1.012)	0.931
		MR Egger	4	1.008 (0.986–1.030)	0.559
		Weighted median	4	1.002 (0.990–1.014)	0.738
CES	Hand grip strength (left)	IVW	4	0.996 (0.979–1.012)	0.609
		MR Egger	4	1.018 (0.998–1.038)	0.219
		Weighted median	4	1.004 (0.993–1.015)	0.527
CES	Hand grip strength (right)	IVW	4	0.986 (0.971–1.001)	0.059
		MR Egger	4	1.004 (0.983–1.026)	0.749
		Weighted median	4	0.991 (0.980–1.002)	0.119
CES	Usual walking pace	IVW	4	0.989 (0.980–0.998)	0.013
		MR Egger	4	0.995 (0.975–1.015)	0.648
		Weighted median	4	0.986 (0.977–0.996)	0.005

**Table 5:** Sensitivity analysis of IS to sarcopenia.

Exposure	Outcome	IVW		MR Egger				MR-PRESSO *P*-value
		Cochran’s *Q* test	*P*-value	Cochran’s *Q* test	*P*-value	Intercept	Intercept *P*-value	
AIS	ALM	9.988	0.125	7.794	0.168	−0.009	0.287	0.153
	Hand grip strength (left)	4.406	0.398	4.055	0.256	4.771E-04	0.944	0.471
	Hand grip strength (right)	6.224	0.183	4.392	0.222	0.007	0.345	0.240
	Usual walking pace	1.297	0.730	1.287	0.525	0.001	0.928	0.746
LAS	ALM	1.808	0.405	0.005	0.946	0.006	0.407	NA
	Hand grip strength (left)	0.366	0.545	NA	NA	NA	NA	NA
	Hand grip strength (right)	1.402	0.236	NA	NA	NA	NA	NA
	Usual walking pace	0.200	0.655	NA	NA	NA	NA	NA
CES	ALM	0.985	0.805	0.417	0.812	−0.002	0.530	0.845
	Hand grip strength (left)	10.982	0.012	2.598	0.273	−0.006	0.126	0.094
	Hand grip strength (right)	8.611	0.035	2.991	0.224	−0.005	0.192	0.157
	Usual walking pace	4.414	0.220	3.554	0.169	−0.002	0.559	0.340

### 3.3 Enrichment analyses

**Figure 4:**
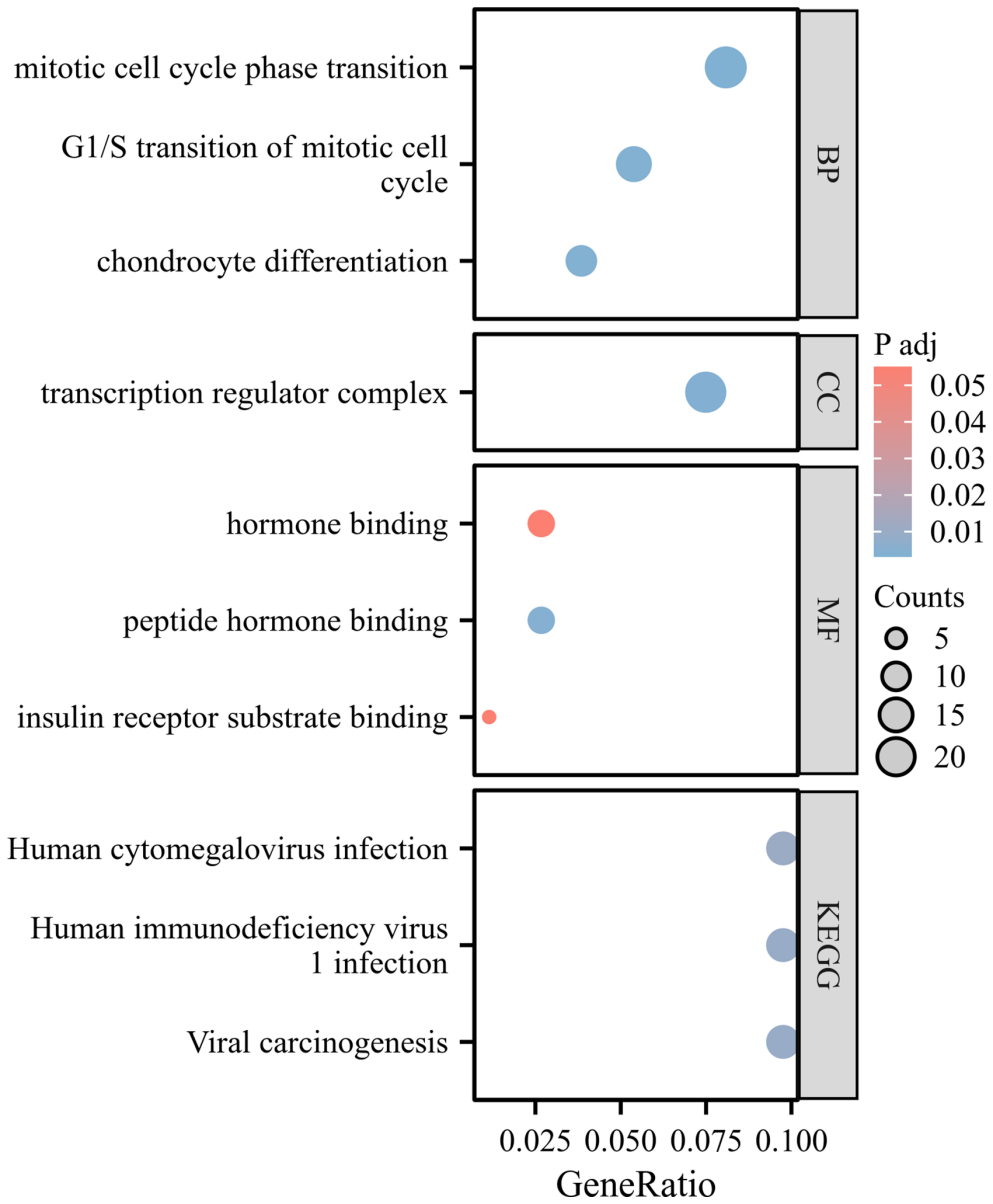
A bubble chart of GO and KEGG enrichment analysis.

During the enrichment analysis between ALM and CES, right-hand grip strength and SVS, as well as between CES and walking speed, we identified a limited number of significant pathways. As a result, we focused the GO and KEGG enrichment analyses on the SNPs identified by ALM and SVS. The corresponding gene names are listed in Supplementary Table S11. GO analysis revealed that the mitotic cell cycle phase transition, G1/S transition of the mitotic cell cycle, and chondrocyte differentiation are associated with Molecular Function (MF). The transcription regulator complex is linked to Cellular Component (CC), while insulin receptor substrate binding and peptide hormone binding are related to Biological Process (BP). KEGG analysis indicates significant enrichment in pathways related to Human cytomegalovirus infection, Human immunodeficiency virus, and Viral carcinogenesis (Figure 4).

## 4 Discussion

The study assessed the bidirectional causal relationship between sarcopenia and ischemic stroke (IS) using two-sample MR analysis. The results indicated that increased ALM was associated with a reduced risk of SVS but an elevated risk of CES. An inverse causal relationship was found between right-hand grip strength and SVS. Reverse MR analysis identified a correlation solely between CES and usual walking pace.

Sarcopenia and IS are prevalent conditions among the elderly, and some studies have identified a correlation between the two. A longitudinal study conducted over 3.6 years, which included approximately 15,000 Chinese individuals, revealed that after adjusting for confounding factors, the risk of stroke was significantly higher in patients with probable sarcopenia (HR = 1.59, 95% CI: 1.26–2.00, *p* < 0.001) or confirmed sarcopenia (HR = 1.67, 95% CI: 1.17–2.40, *p* < 0.01) (5). Furthermore, a meta-analysis involving 63,738,162 participants supported these findings, indicating an increased stroke risk in sarcopenia patients (OR = 1.67, 95% CI: 1.18–2.37, *p* = 0.004) (7). However, these studies did not examine the influence of sarcopenia on specific stroke subtypes. To address this gap, we expanded upon previous research by utilizing MR analysis to assess the causal effect of genetically predicted sarcopenia on different stroke subtypes.

Inflammation, metabolic disorders, physical inactivity associated with a sedentary lifestyle, hormonal changes related to aging, and endothelial dysfunction collectively contribute to the connection between sarcopenia and stroke. A sedentary lifestyle exacerbates metabolic disorders, endothelial dysfunction, and inflammation levels (31, 32). Insulin resistance and endothelial dysfunction have a reciprocal relationship (33), while the decline in testosterone and estrogen with age further accelerates endothelial dysfunction (34, 35). The interaction of these factors increases the risk of stroke. Sarcopenia is marked by a pro-inflammatory state driven by cytokines and oxidative stress (36), which also plays a significant pathogenic role in stroke (37, 38). Skeletal muscles secrete myokines in response to physical activity, which have anti-inflammatory effects (39). Enhancing muscle mass can promote myokine secretion to combat inflammation (40, 41). An eight-year study demonstrated a negative correlation between grip strength and inflammation (42), and another study found that individuals with low grip strength had elevated C-reactive protein (CRP) levels (OR = 1.60, 95% CI = 1.03–2.49) (43). CRP, as an inflammation marker, is closely associated with stroke risk. This research helps explain how the decline in muscle mass and strength may increase stroke risk through inflammatory pathways. However, our study suggests a more pronounced relationship between muscle issues and SVS, possibly due to inadequate collateral circulation in small arteries.

Additionally, research indicates that sarcopenia is an aging-related syndrome closely associated with immune function (44). This may suggest an increased risk of viral infections in elderly patients (45). Chronic or acute stimulation of the immune system by viral exposure can lead to coagulation dysfunction and subsequently induce IS (46–48). The GO and KEGG enrichment analysis in this study corroborate this finding.

We identified a positive correlation between ALM and CES using MR, which contrasts with the findings for SVS. Causes of CES include atrial fibrillation, heart failure, coronary artery disease, cardiomyopathy, valvular heart disease, and other related conditions (49). To date, only a few clinical studies have examined the relationship between muscle mass and these conditions (50–52), and no studies have specifically addressed CES. Based on our findings, one possible mechanism involves iron metabolism. Previous research has shown that reduced muscle mass is associated with iron deficiency (53). Chang et al. reported that individuals with thrombotic and embolic strokes had a higher incidence of prior iron deficiency diagnoses compared to healthy controls (54). However, this alone does not fully explain the paradoxical result. Enrichment analysis also failed to uncover an effective biological mechanism. Considering that CES primarily results from cardiac conditions, whereas SVS is caused by intrinsic lesions in small cerebral vessels, the differing etiologies may account for the observed outcome variations. Future research should explore the mechanisms linking muscle mass to IS subtypes.

Stroke can lead to systemic muscle mass loss and functional decline through various mechanisms, including prolonged bed rest, motor function loss, feeding difficulties, poor nutritional status, sympathetic activation, inflammatory responses, and nerve damage. These factors contribute to the onset and progression of sarcopenia, thereby increasing the overall burden on patients (55–58). The incidence of gait disorders due to neurological deficits from stroke is notably high and is typically characterized by reduced stride length, decreased walking speed, and impaired balance control. These impairments contribute to difficulties in daily activities and an increased risk of falls (59). Additionally, studies have shown that heart disease is associated with decreased walking speed (60, 61). In our MR study, cardiac causes of CES were found to be negatively correlated with usual walking pace, suggesting that these factors may synergistically contribute to gait disorders. However, due to the limited number of SNPs identified for stroke subtypes in our reverse MR analysis, our results have certain limitations. While our study found a significant relationship between CES and walking speed, the causal effect derived from the limited IVs may be relatively weak. Therefore, future studies should analyze and interpret causal relationships using larger GWAS datasets.

The MR study initially explored the causal relationships between sarcopenia-related traits and IS subtypes, yielding positive findings. Compared to traditional observational studies, MR research minimizes the influence of confounding factors, providing more robust results by accounting for heterogeneity and horizontal pleiotropy. However, several limitations exist. Firstly, unknown confounding factors may still be present, potentially influencing the outcomes. Secondly, the study population was exclusively of European descent, which limits the generalizability of the findings to other populations. Lastly, the proportion of IS subtypes varies across different racial groups, indicating the need for further studies to investigate causal effects in diverse populations.

## 5 Conclusion

In summary, our research utilized large-scale gene summary data to identify a bidirectional causal relationship between sarcopenia and IS. Specifically, sarcopenia indicators, such as ALM and right-hand grip strength, were negatively correlated with SVS, while ALM and CES assessment showed a positive correlation. Additionally, CES assessment was negatively correlated with usual walking pace. To further investigate the complex relationship between sarcopenia and stroke, additional studies involving diverse populations and experimental research are required to clarify the underlying mechanisms and improve prevention and treatment strategies.

## Data Availability

The datasets presented in this study can be found in online repositories. The names of the repository/repositories and accession number(s) can be found in the article/Supplementary material.
