# Supplementary material for: Ischemic stroke and sarcopenia have an asymmetric bidirectional relationship based on a two-sample Mendelian randomization study
Source: Front Neurol. 2024 Oct 9;15:1427692. doi: 10.3389/fneur.2024.1427692 (PMC11499911; doi:10.3389/fneur.2024.1427692)
Supplement: Supplementary file 1 [file Data_Sheet_1.docx]

**Supplementary Figures**

Supplementary Figure 1: Funnel plot of ALM on IS and subtypes

Supplementary Figure 2: Funnel plot of Hand grip strength (left) on IS and subtypes

Supplementary Figure 3: Funnel plot of Hand grip strength (right) on IS and subtypes

Supplementary Figure 4: Funnel plot of Usual walking pace on IS and subtypes

Supplementary Figure 5: Funnel plot of AIS on sarcopenia-related traits

Supplementary Figure 6: Funnel plot of LAS on sarcopenia-related traits

Supplementary Figure 7: Funnel plot of CES on sarcopenia-related traits

Supplementary Figure 8: Scatter plot of ALM on IS and subtypes

Supplementary Figure 9: Scatter plot of Hand grip strength (left) on IS and subtypes

Supplementary Figure 10: Scatter plot of Hand grip strength (right) on IS and subtypes

Supplementary Figure 11: Scatter plot of Usual walking pace on IS and subtypes

Supplementary Figure 12: Scatter plot of AIS on sarcopenia-related traits

Supplementary Figure 13: Scatter plot of LAS on sarcopenia-related traits

Supplementary Figure 14: Scatter plot of CES on sarcopenia-related traits

Supplementary Figure 15: Leave-one-out of Usual walking pace on AIS and CES

Supplementary Figure 16: Leave-one-out of Usual walking pace on LAS and SVS

Supplementary Figure 17: Leave-one-out of IS and subtypes on sarcopenia-related traits

Supplementary Figure 18: Leave-one-out of Hand grip strength (left) on AIS

Supplementary Figure 19: Leave-one-out of Hand grip strength (left) on CES

Supplementary Figure 20: Leave-one-out of Hand grip strength (left) on LAS

Supplementary Figure 21: Leave-one-out of Hand grip strength (left) on SVS

Supplementary Figure 22: Leave-one-out of Hand grip strength (right) on AIS

Supplementary Figure 23: Leave-one-out of Hand grip strength (right) on CES

Supplementary Figure 24: Leave-one-out of Hand grip strength (right) on LAS

Supplementary Figure 25: Leave-one-out of Hand grip strength (right) on SVS

Supplementary Figure 26: Leave-one-out of ALM on AIS

Supplementary Figure 27: Leave-one-out of ALM on CES

Supplementary Figure 28: Leave-one-out of ALM on LAS

Supplementary Figure 29: Leave-one-out of ALM on SVS


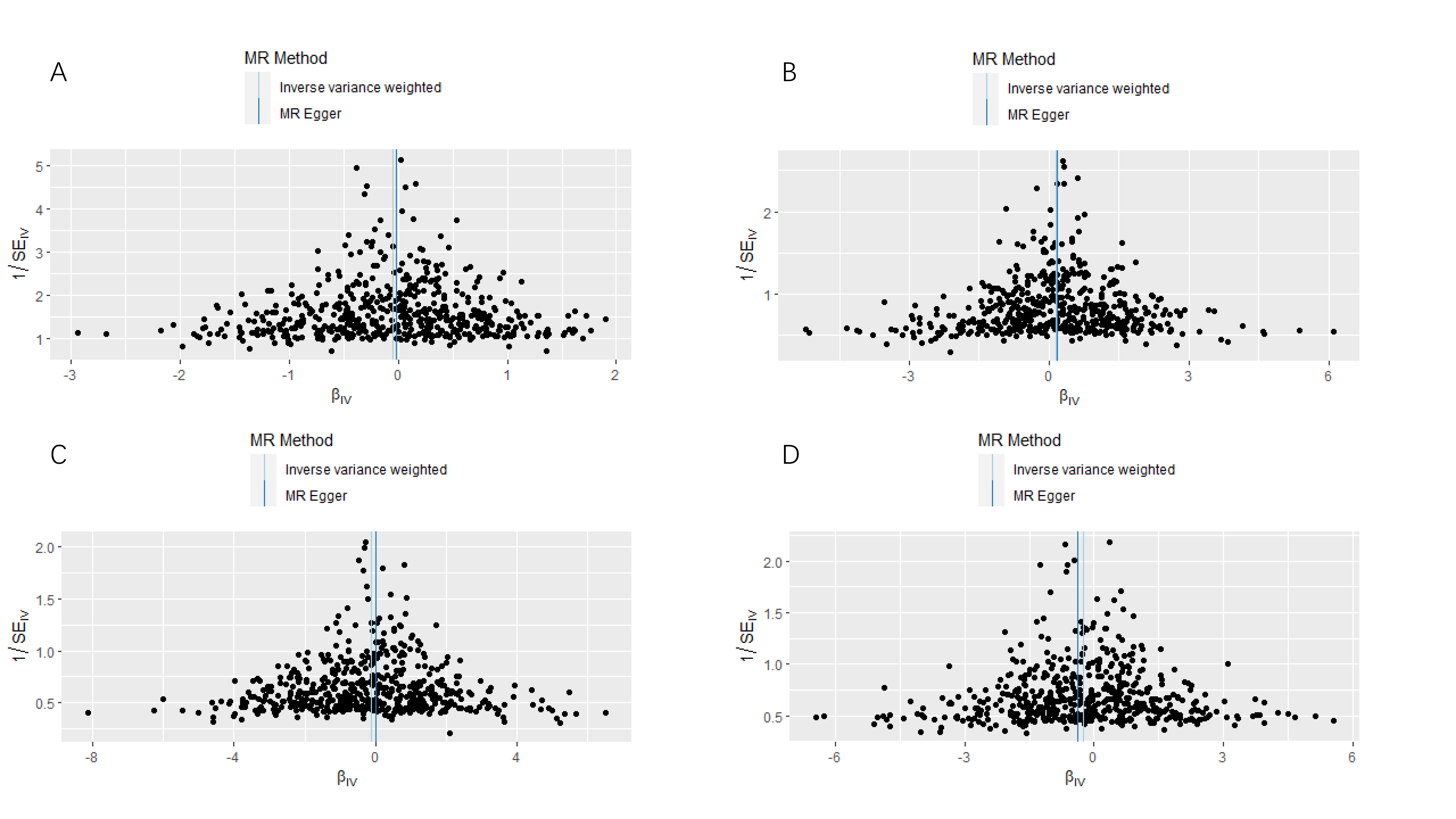


Supplementary Figure 1: (A) ALM on AIS; (B) ALM on CES; (C) ALM on LAS; (D) ALM on SVS.


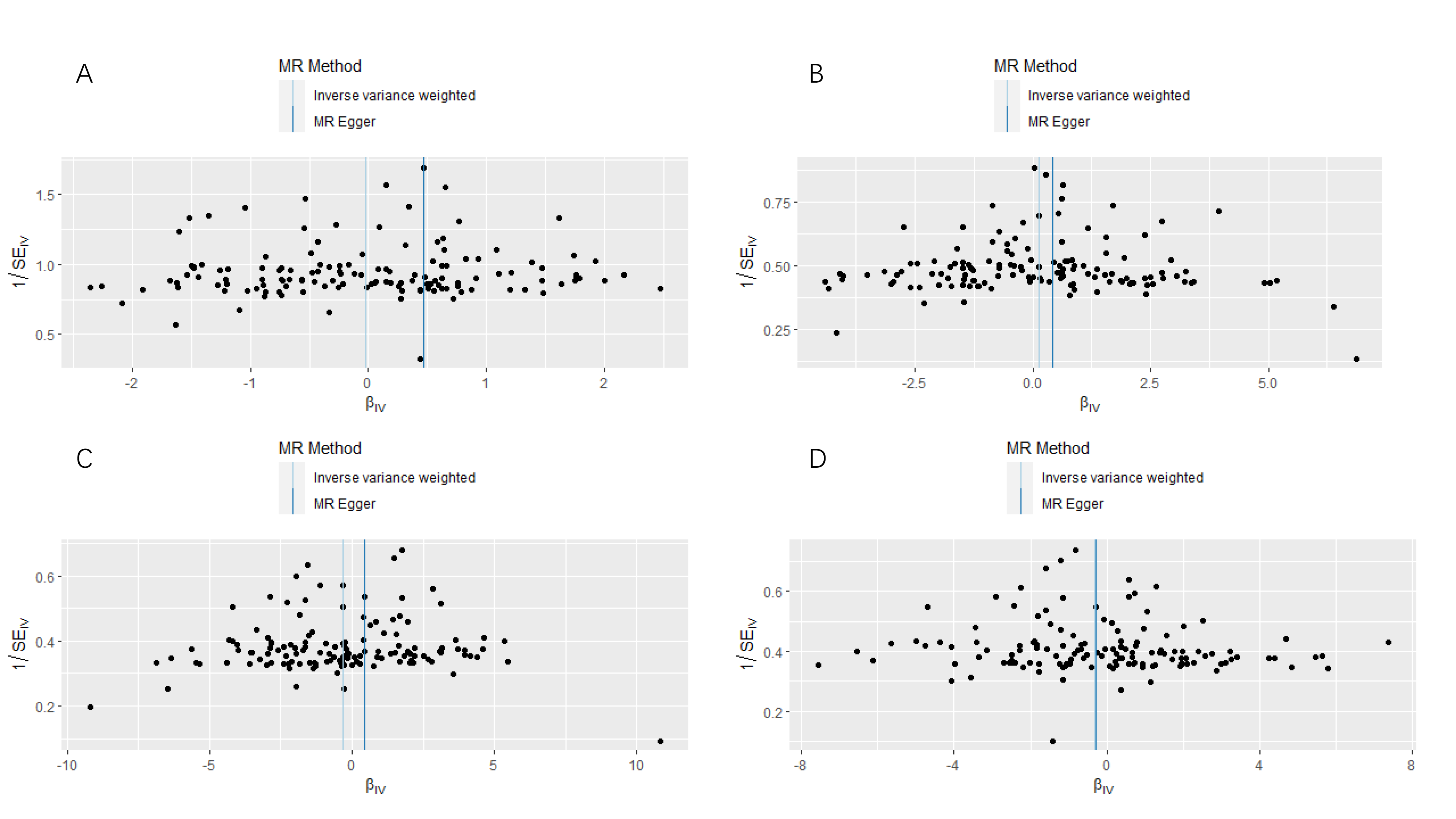


Supplementary Figure 2: (A) Hand grip strength (left) on AIS; (B) Hand grip strength (left) on CES; (C) Hand grip strength (left) on LAS; (D) Hand grip strength (left) on SVS.


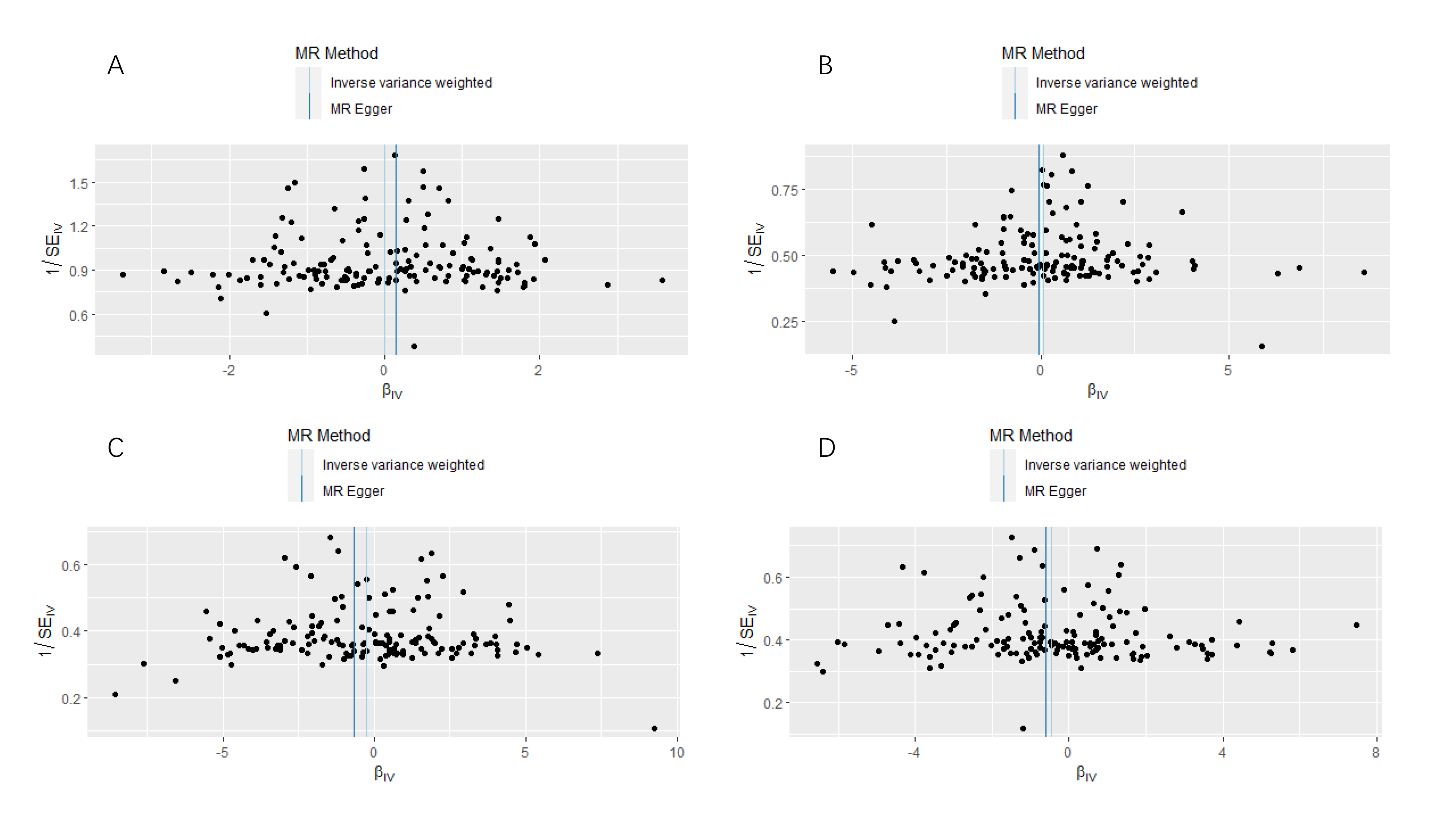


Supplementary Figure 3: (A) Hand grip strength (right) on AIS; (B) Hand grip strength (right) on CES; (C) Hand grip strength (right) on LAS; (D) Hand grip strength (right) on SVS.


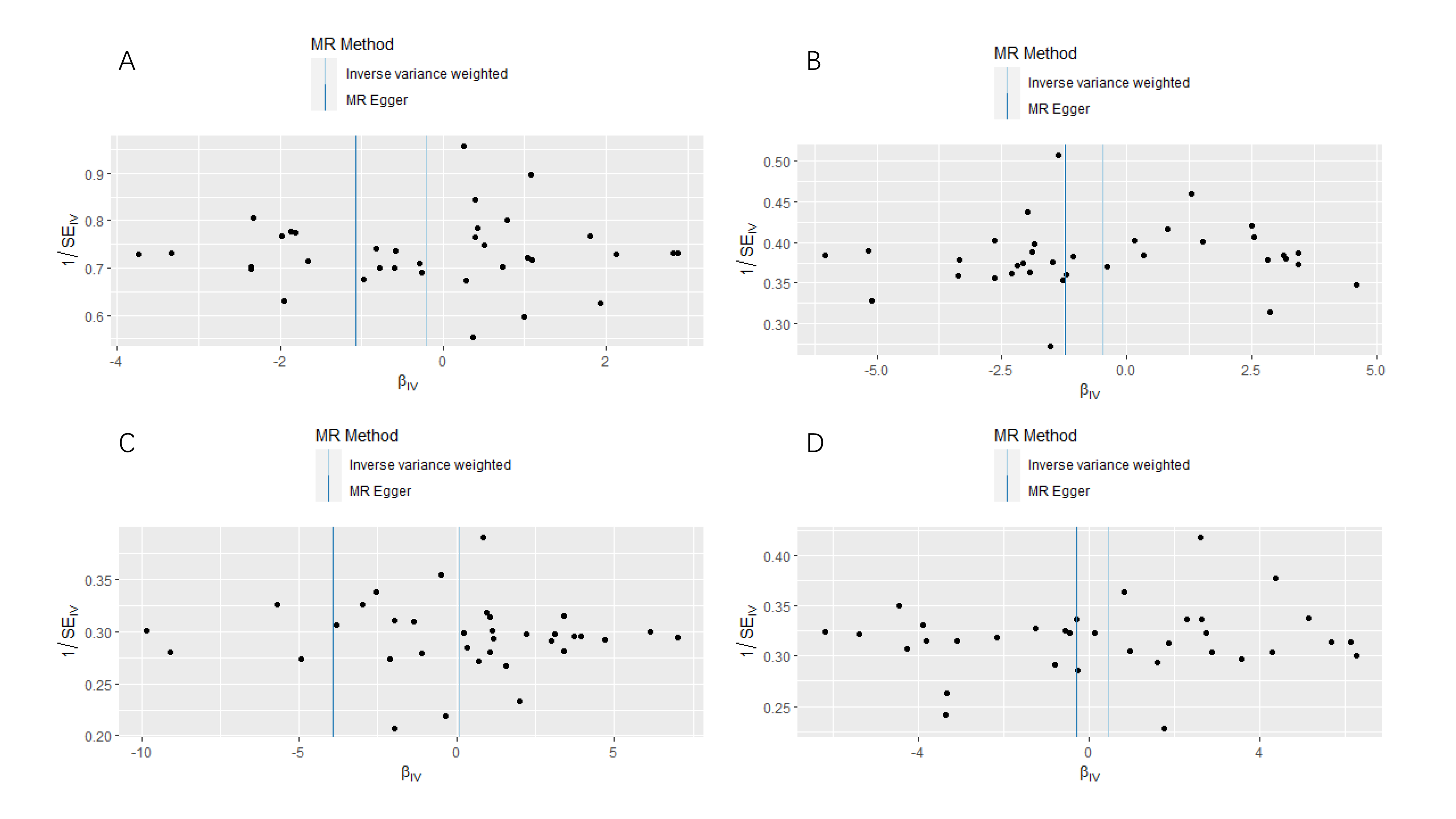


Supplementary Figure 4: (A) Usual walking pace on AIS; (B) Usual walking pace on CES; (C) Usual walking pace on LAS; (D) Usual walking pace on SVS.


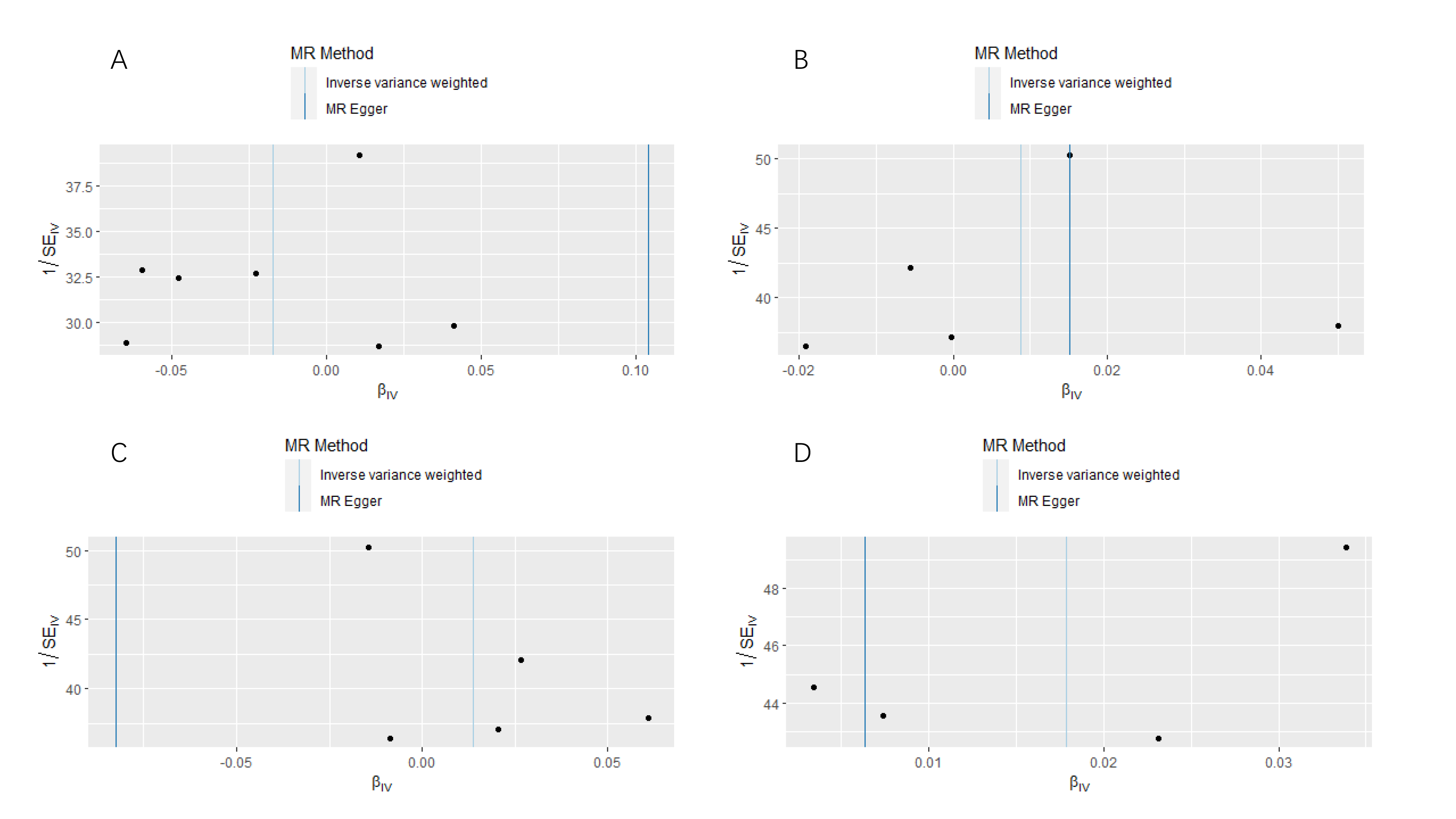


Supplementary Figure 5: (A) AIS on ALM; (B) AIS on Hand grip strength (left); (C) AIS on Hand grip strength (right); (D) AIS on Usual walking pace.


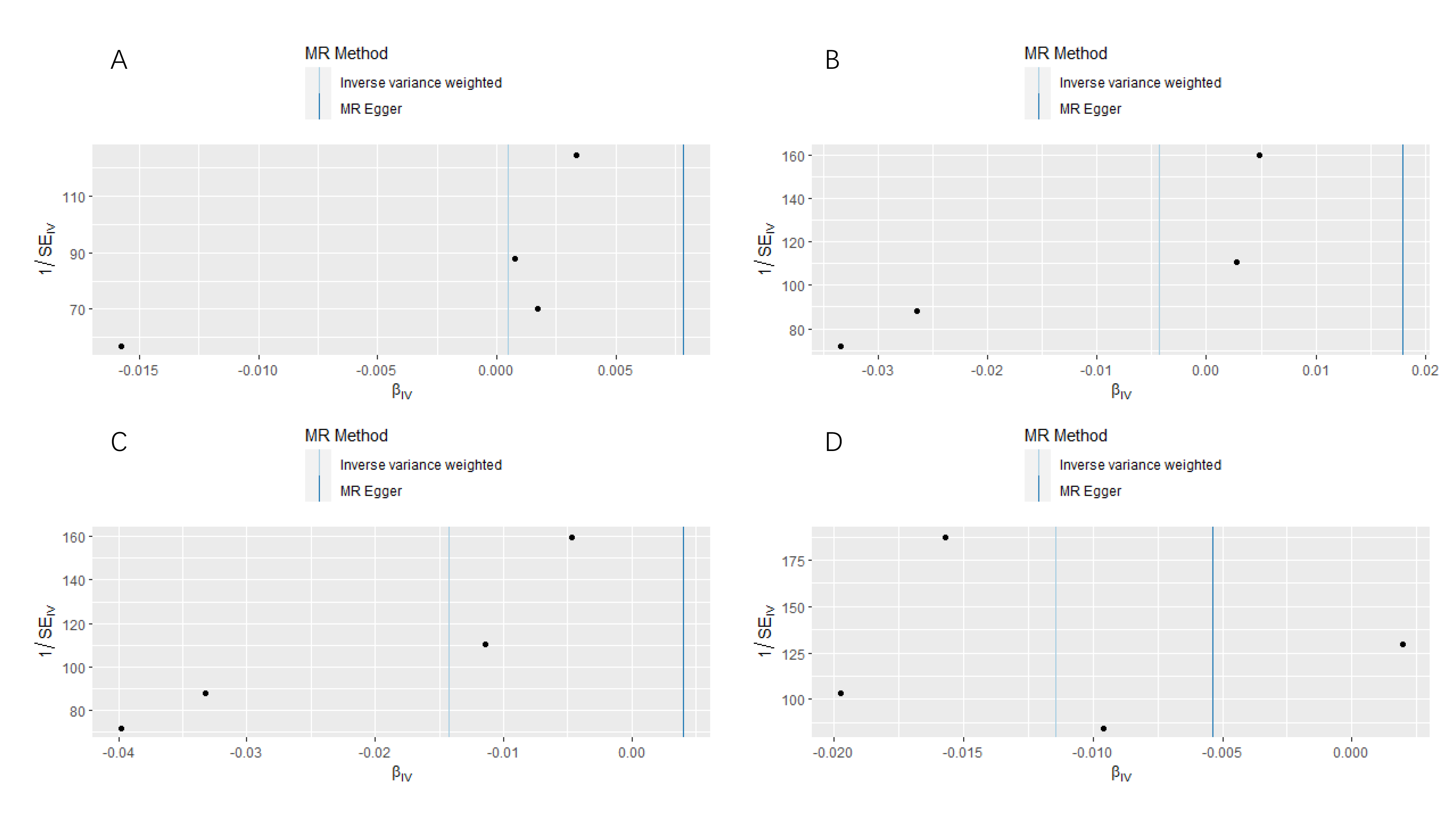


Supplementary Figure 6: (A) CES on ALM; (B) CES on Hand grip strength (left); (C) CES on Hand grip strength (right); (D) CES on Usual walking pace.


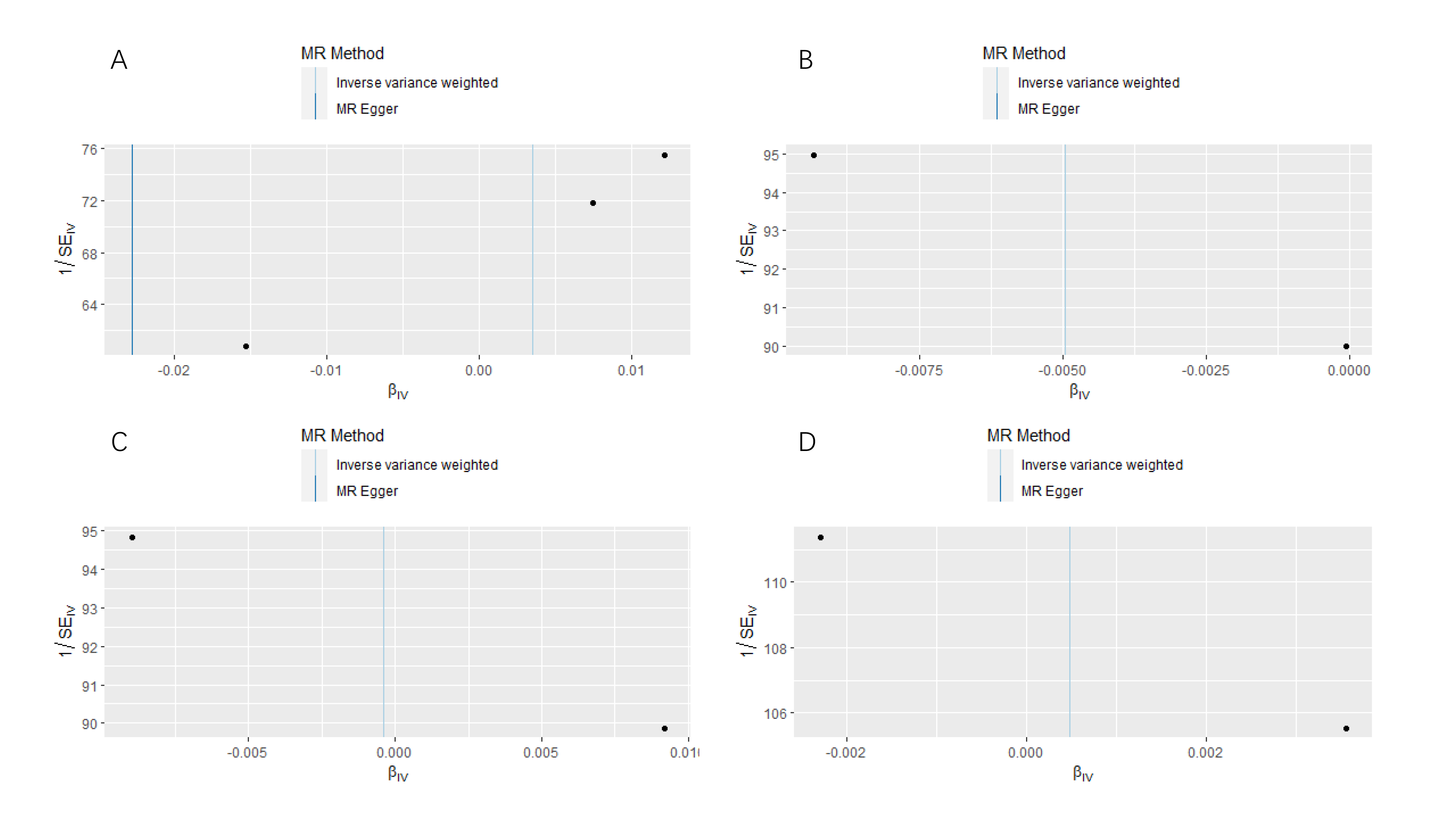


Supplementary Figure 7: (A) LAS on ALM; (B) LAS on Hand grip strength (left); (C) LAS on Hand grip strength (right); (D) LAS on Usual walking pace.


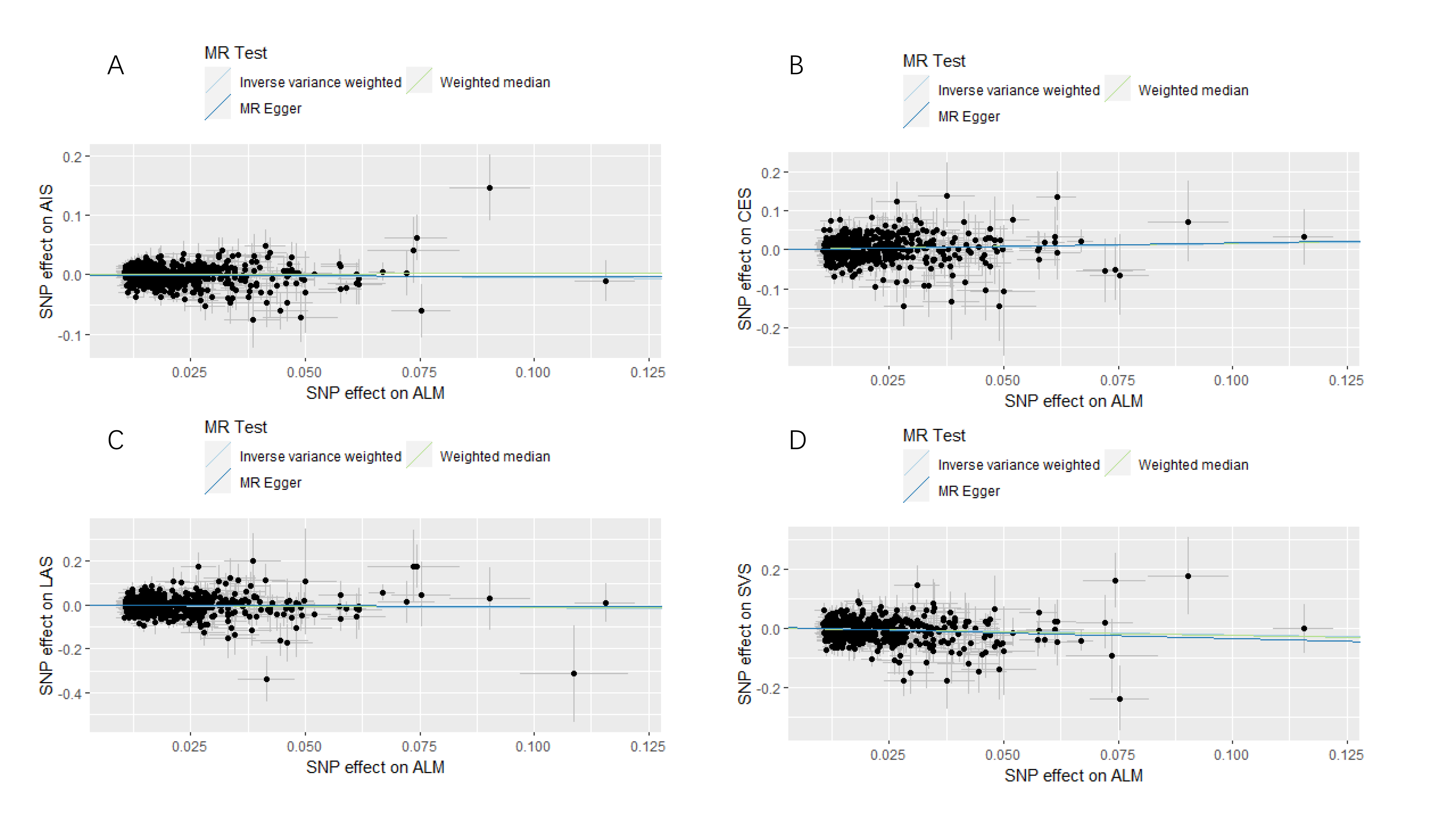


Supplementary Figure 8: (A) ALM on AIS; (B) ALM on CES; (C) ALM on LAS; (D) ALM on SVS.


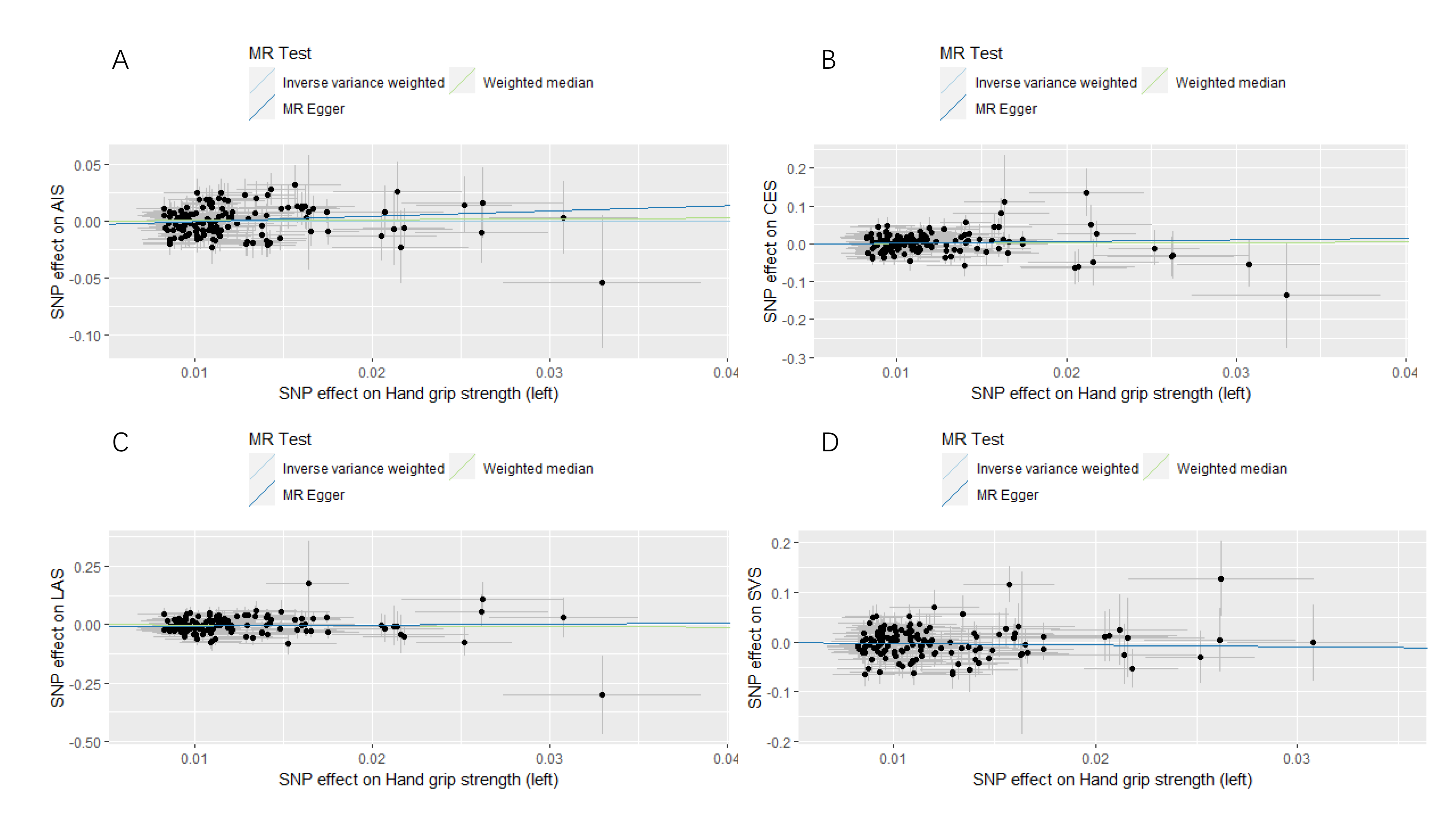


Supplementary Figure 9: (A) Hand grip strength (left) on AIS; (B) Hand grip strength (left) on CES; (C) Hand grip strength (left) on LAS; (D) Hand grip strength (left) on SVS.
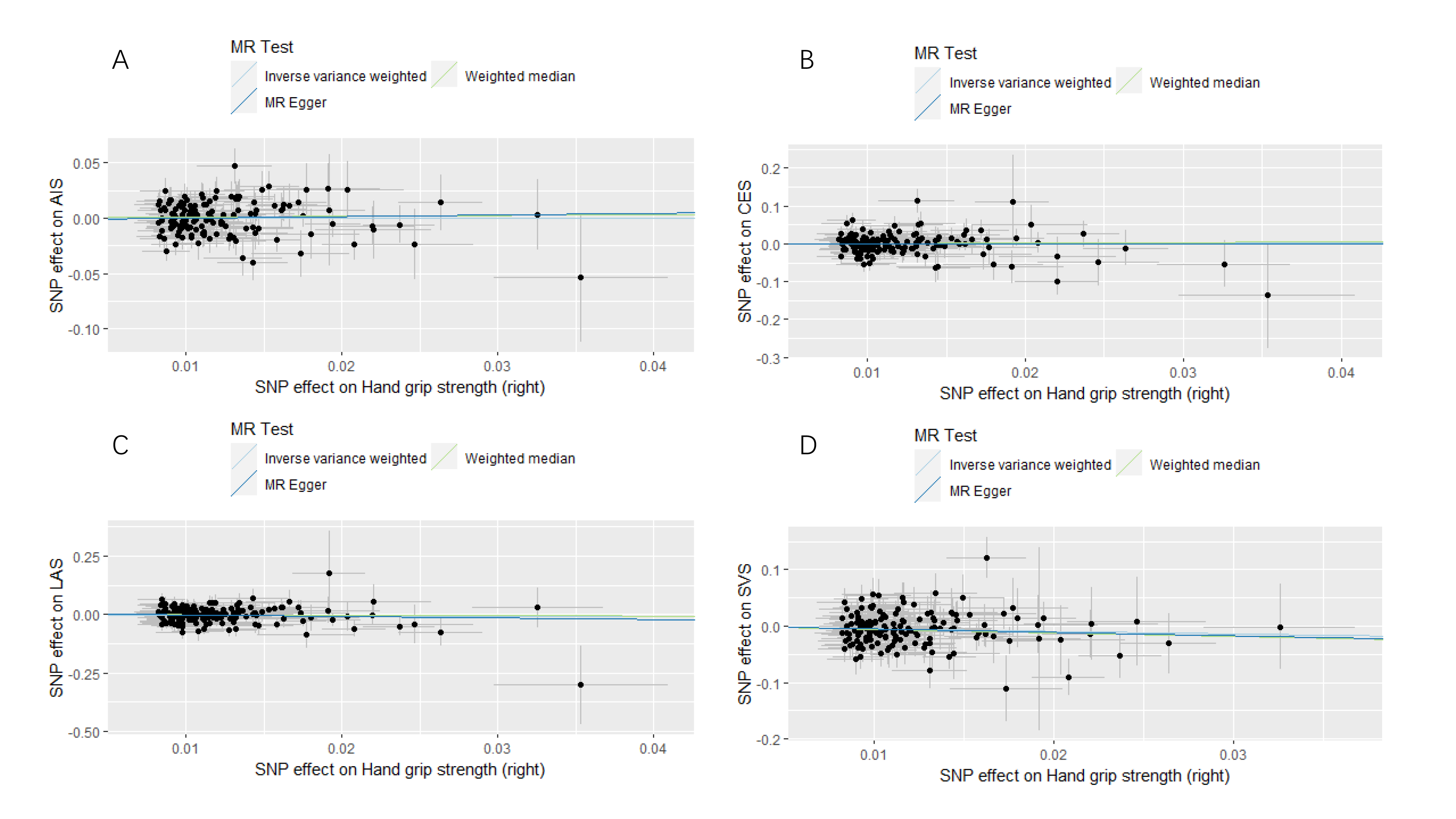


Supplementary Figure 10: (A) Hand grip strength (right) on AIS; (B) Hand grip strength (right) on CES; (C) Hand grip strength (right) on LAS; (D) Hand grip strength (right) on SVS.
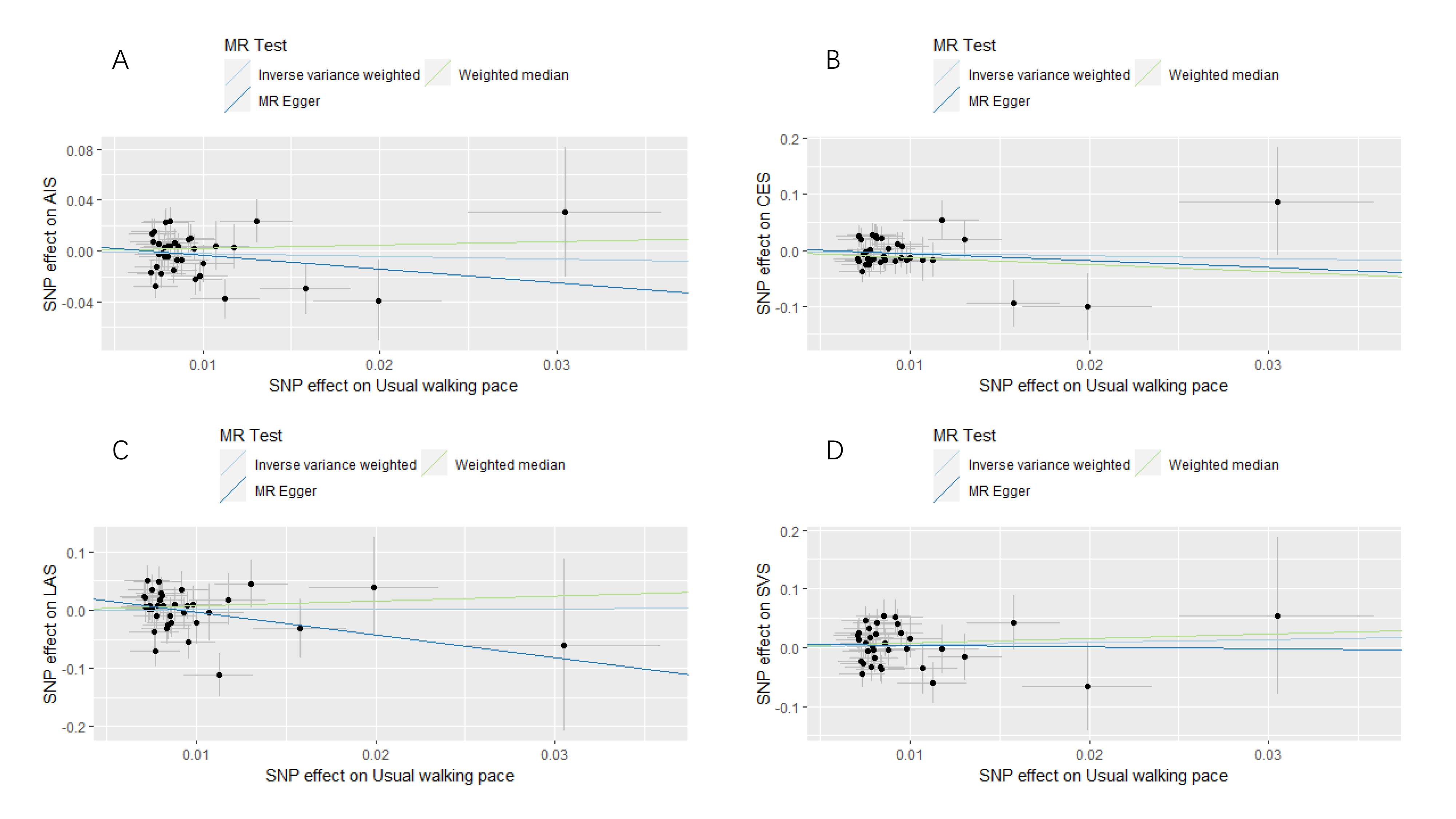


Supplementary Figure 11: (A) Usual walking pace on AIS; (B) Usual walking pace on CES; (C) Usual walking pace on LAS; (D) Usual walking pace on SVS.


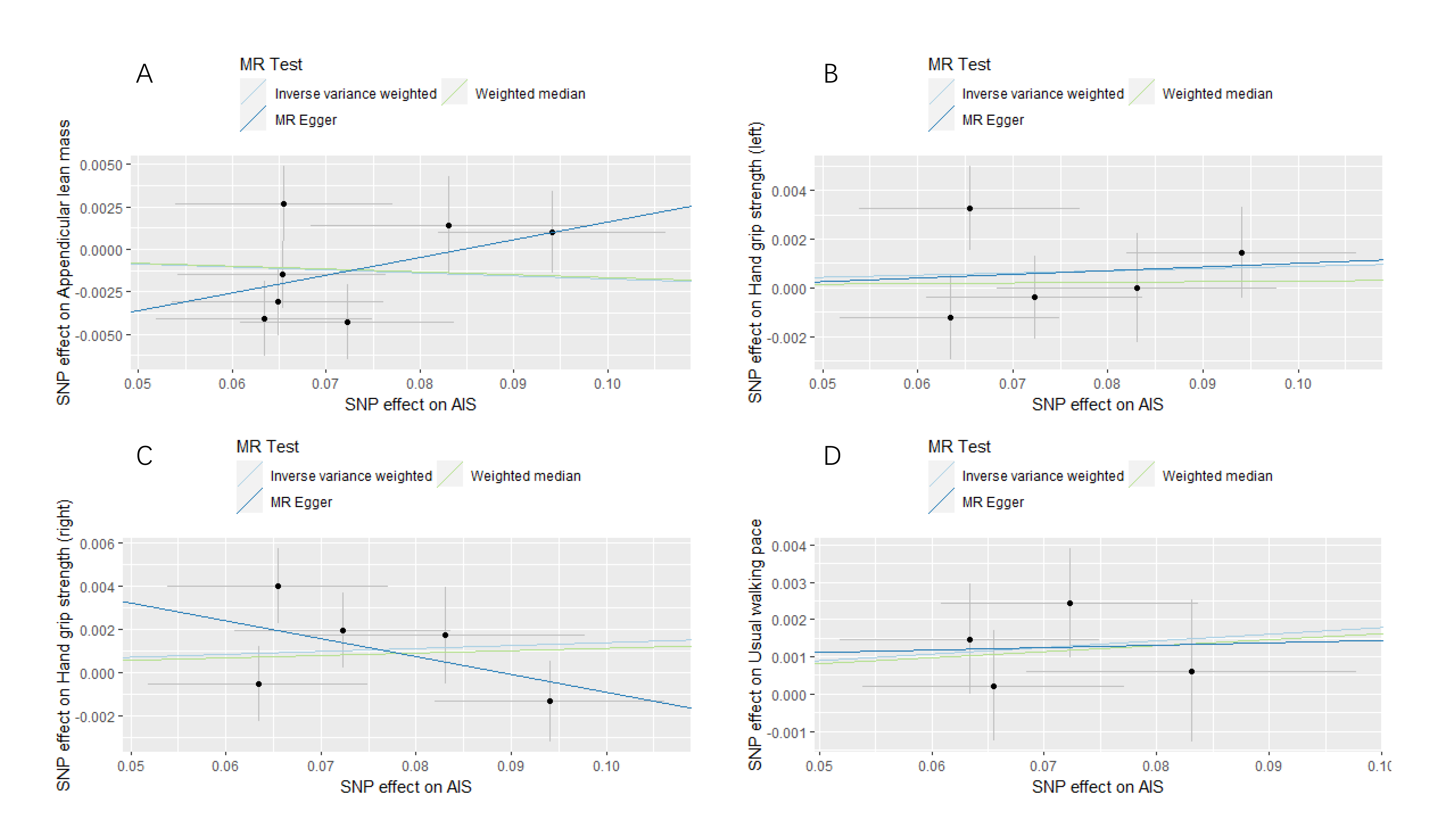


Supplementary Figure 12: (A) AIS on ALM; (B) AIS on Hand grip strength (left); (C) AIS on Hand grip strength (right); (D) AIS on Usual walking pace.


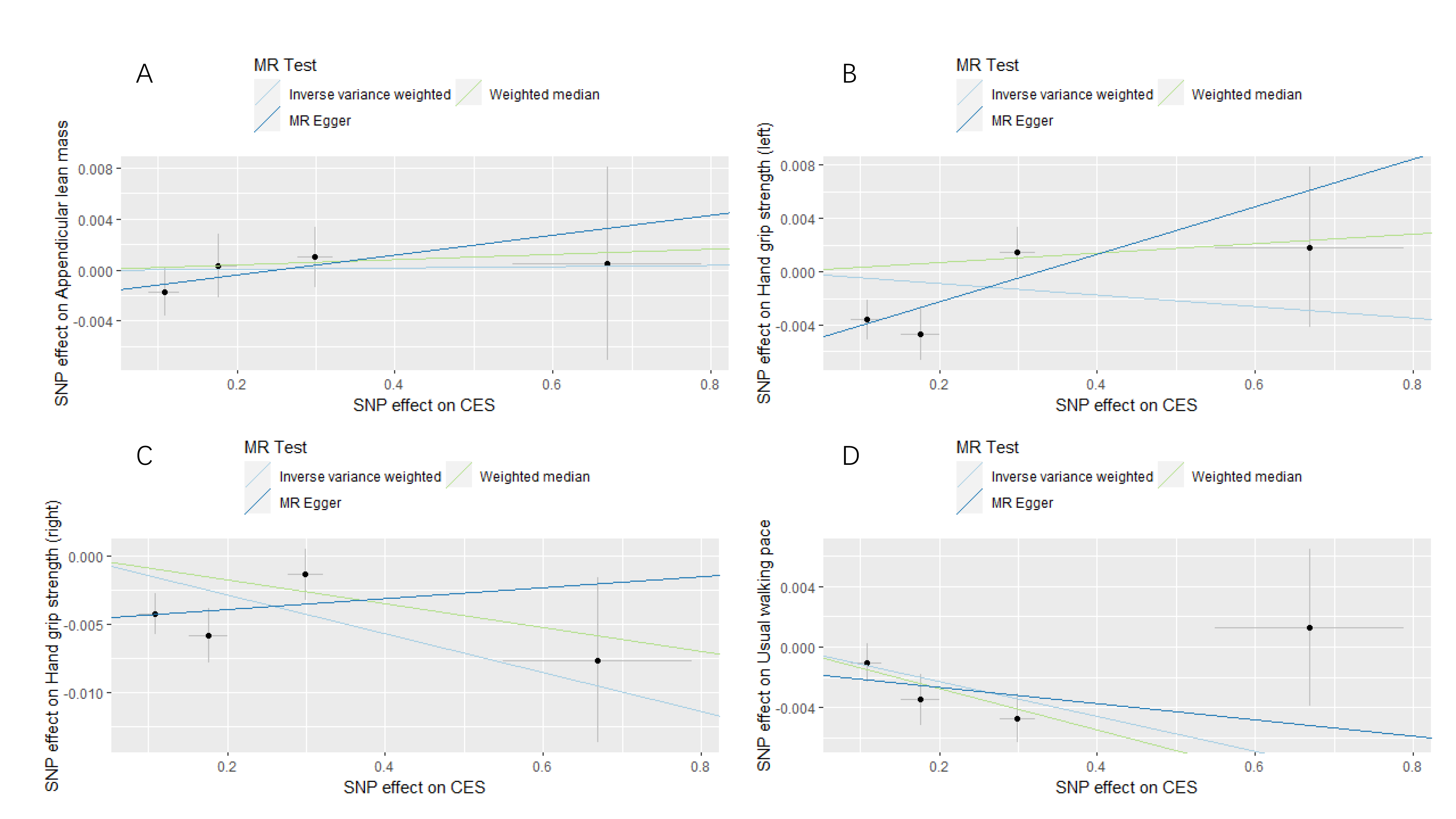


Supplementary Figure 13: (A) CES on ALM; (B) CES on Hand grip strength (left); (C) CES on Hand grip strength (right); (D) CES on Usual walking pace.


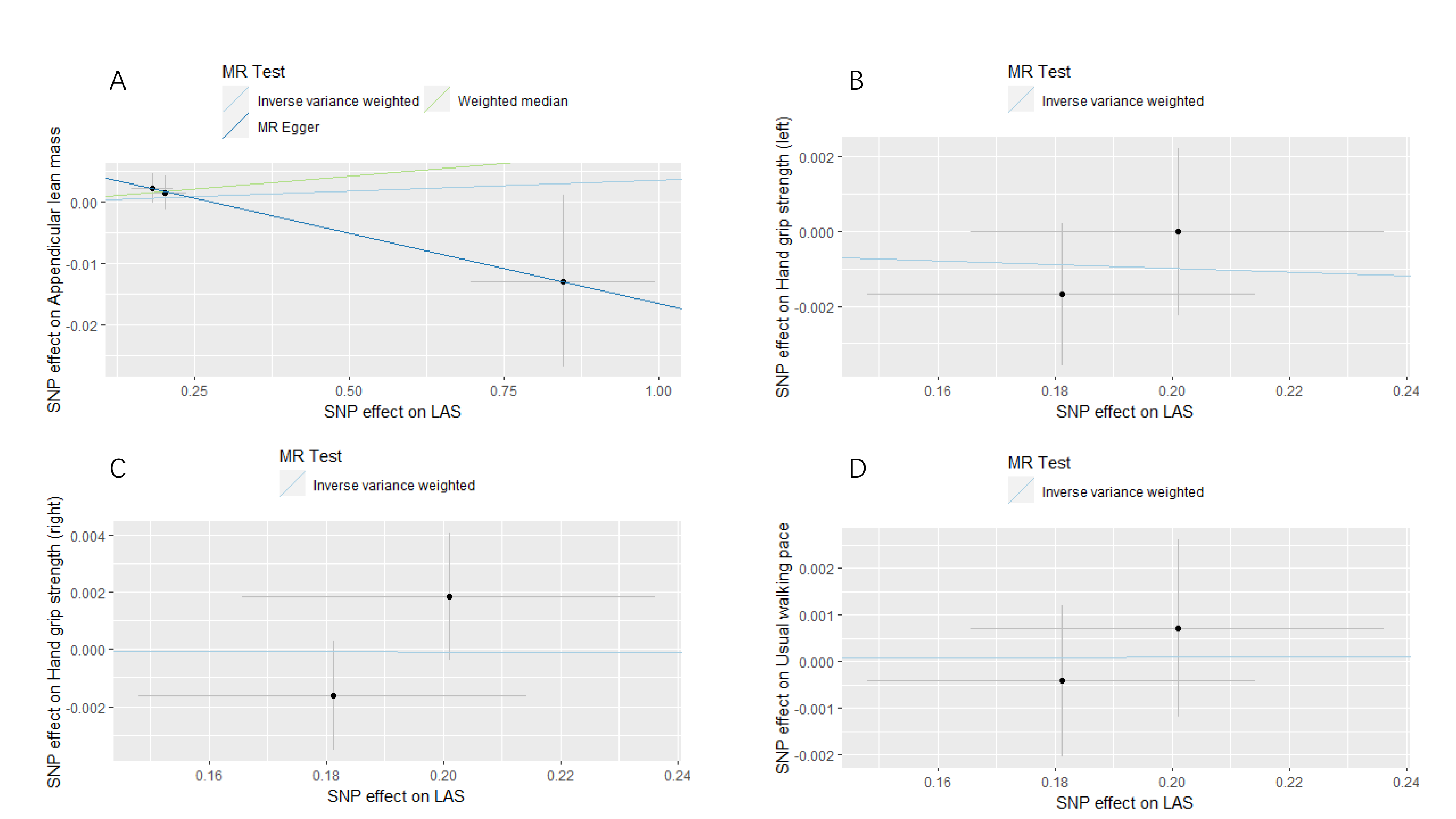


Supplementary Figure 14: (A) LAS on ALM; (B) LAS on Hand grip strength (left); (C) LAS on Hand grip strength (right); (D) LAS on Usual walking pace.
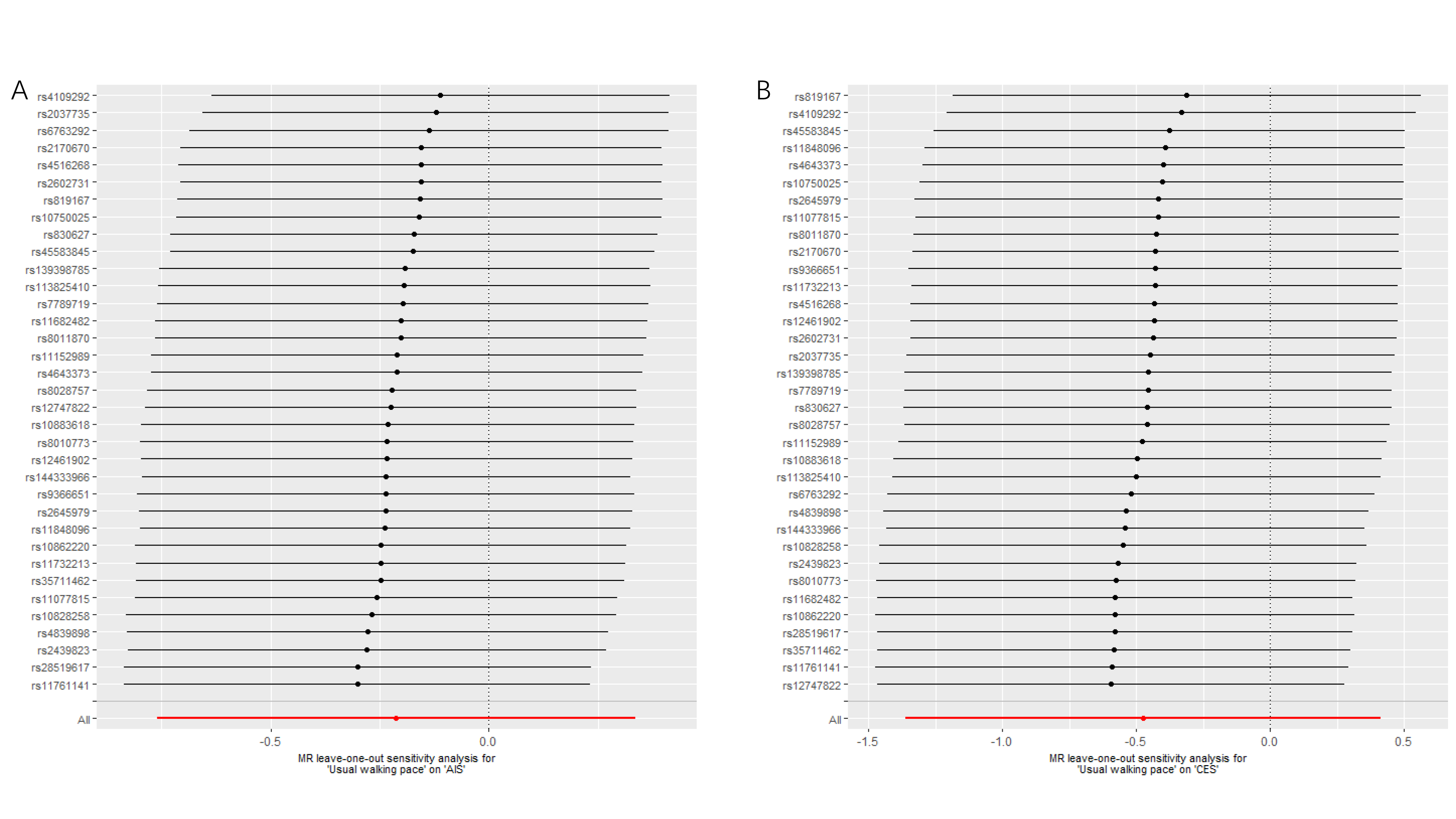


Supplementary Figure 15: (A) Usual walking pace on AIS; (B) Usual walking pace on CES.


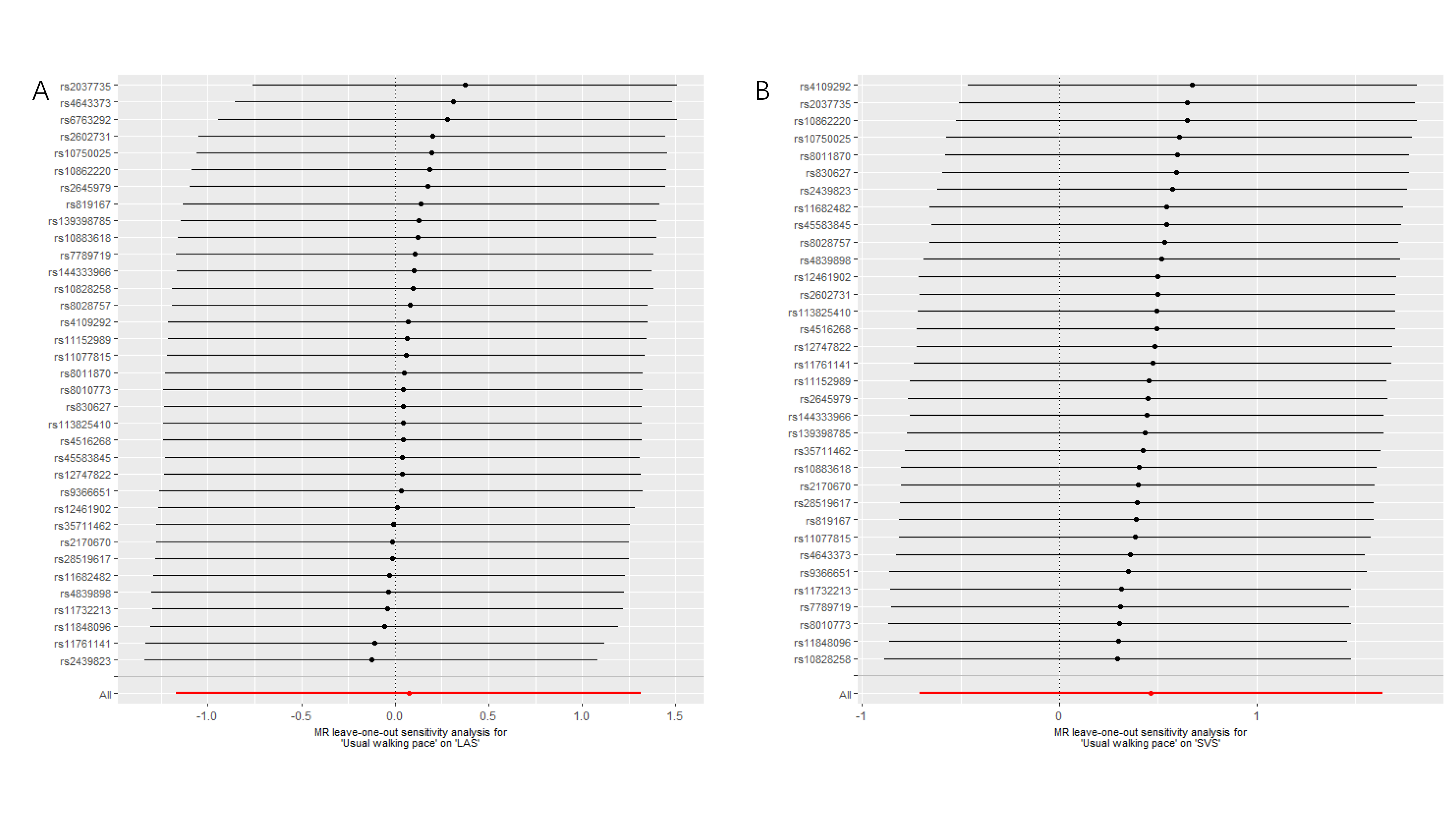


Supplementary Figure 16: (A) Usual walking pace on LAS; (B) Usual walking pace on SVS.


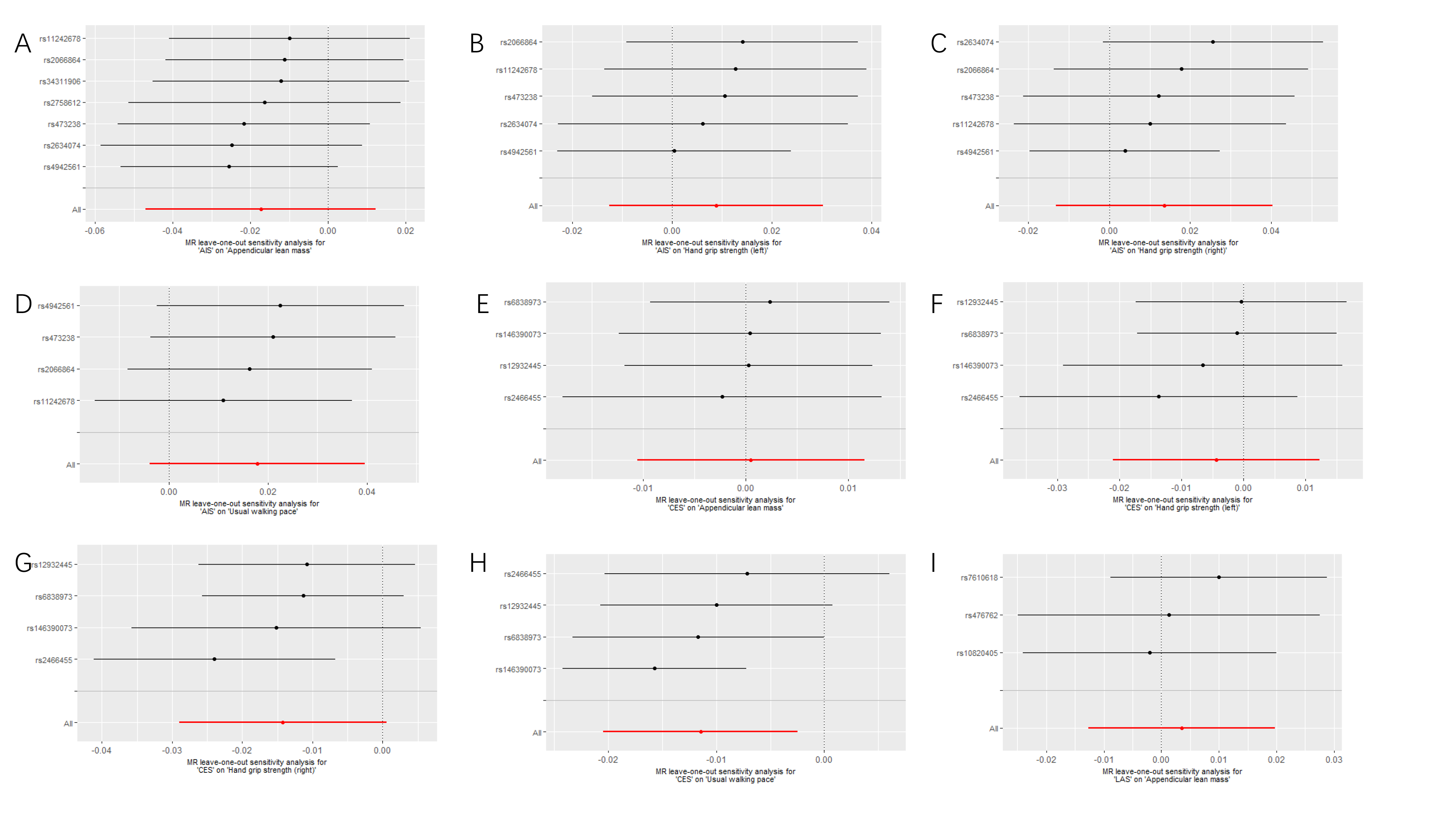


Supplementary Figure 17: (A) AIS on ALM; (B) AIS on Hand grip strength (left); (C) AIS on Hand grip strength (right); (D) AIS on Usual walking pace; (E) CES on ALM; (F) CES on Hand grip strength (left); (G) CES on Hand grip strength (right); (H) CES on Usual walking pace; (I) LAS on ALM.
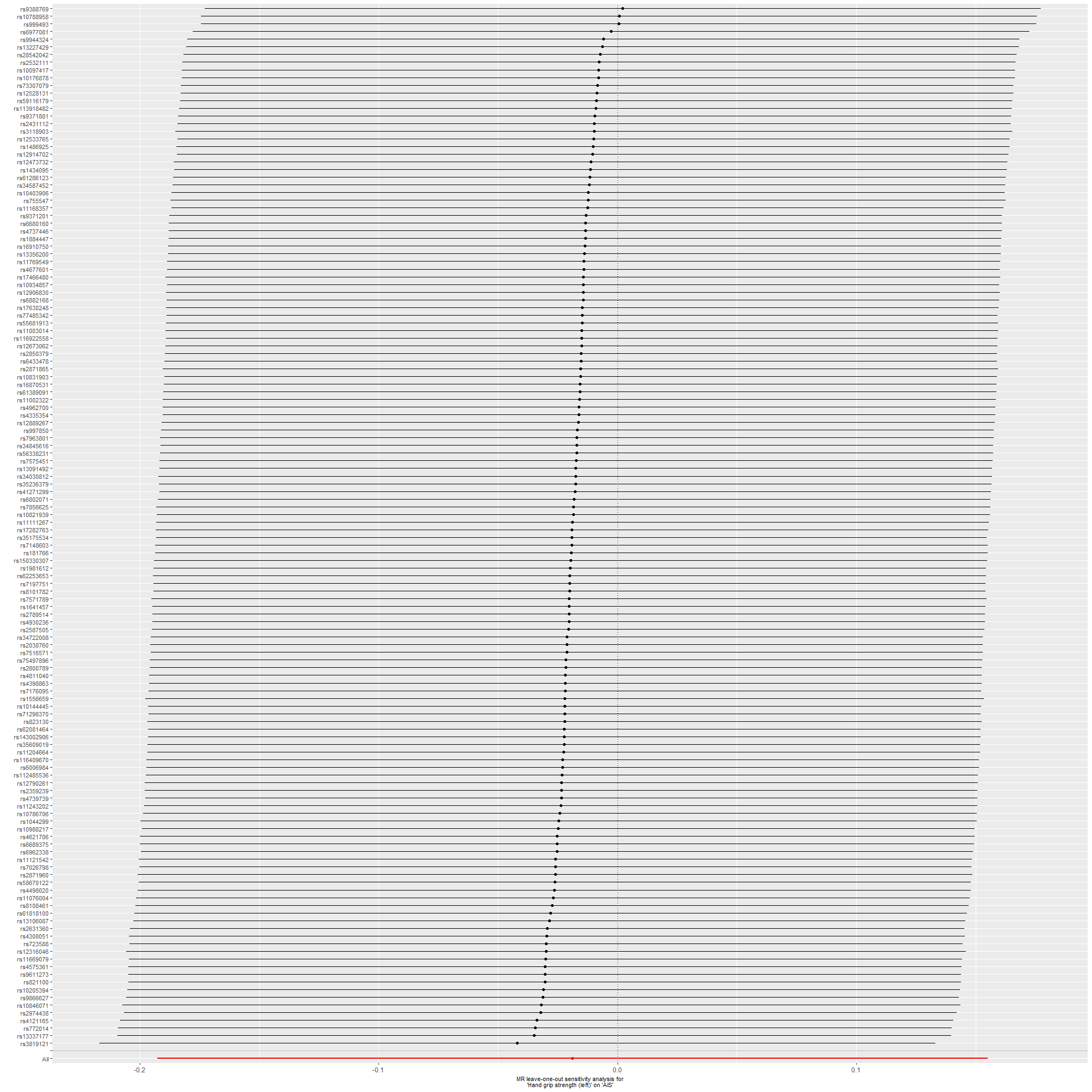


Supplementary Figure 18: Hand grip strength (left) on AIS.
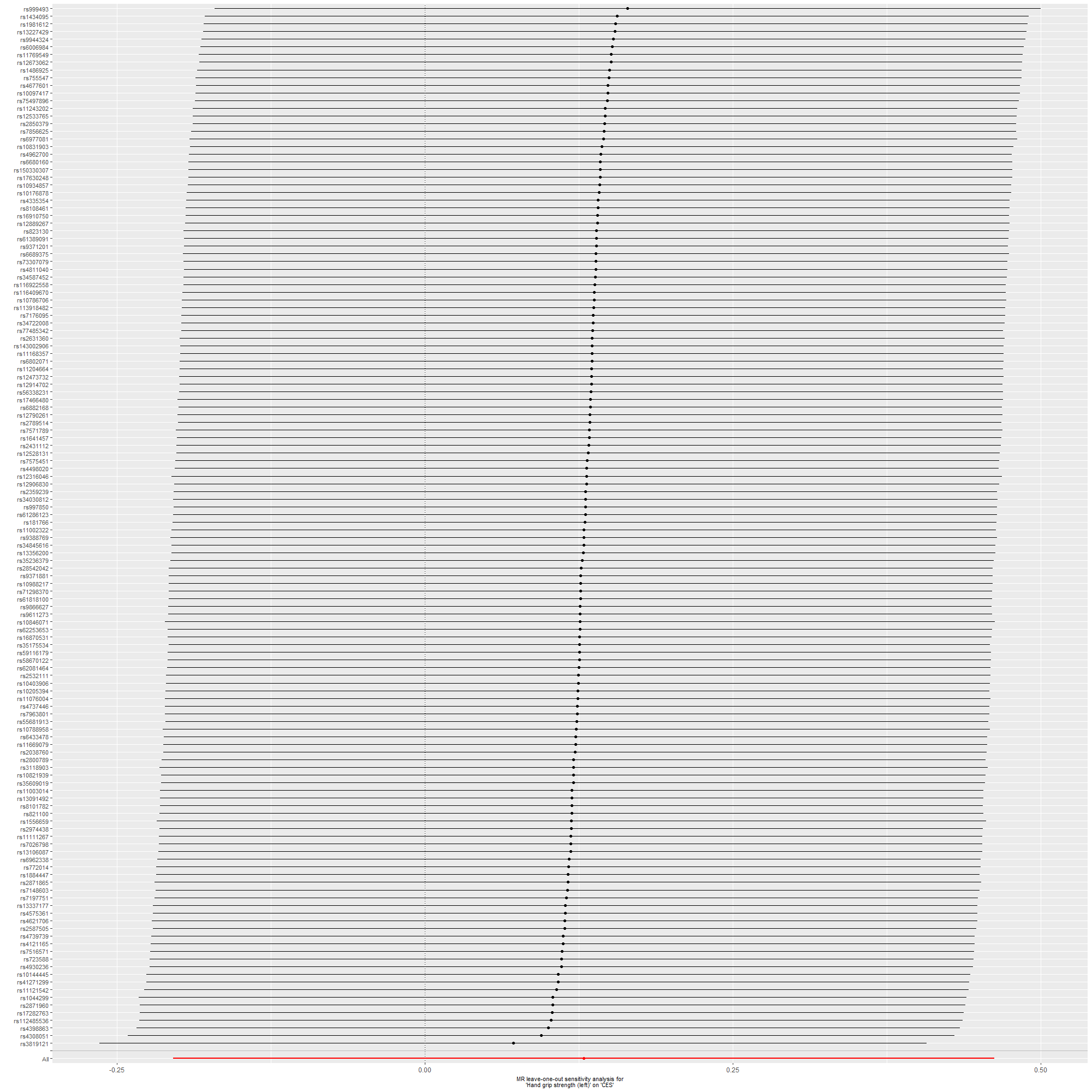


Supplementary Figure 19: Hand grip strength (left) on CES.
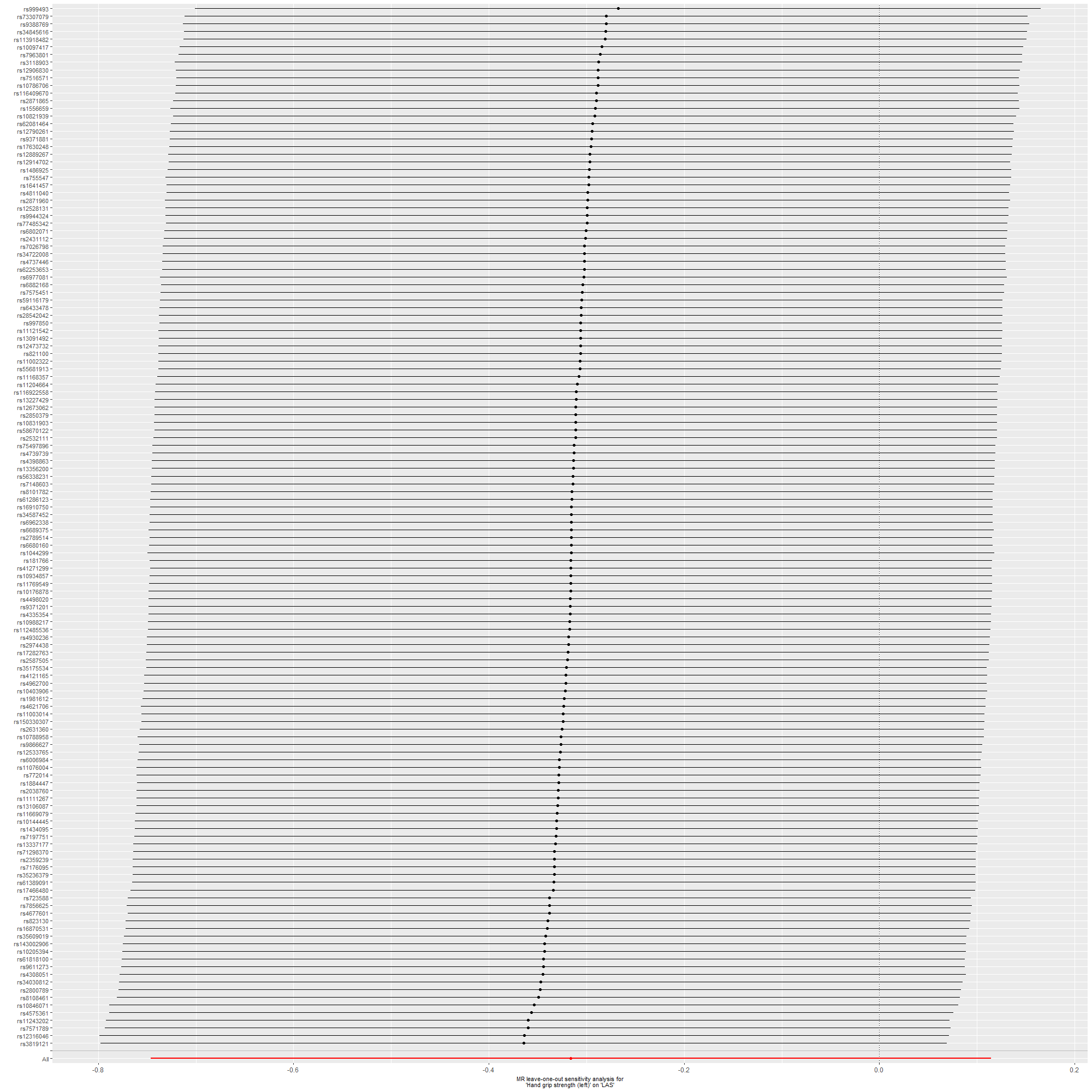


Supplementary Figure 20: Hand grip strength (left) on LAS.
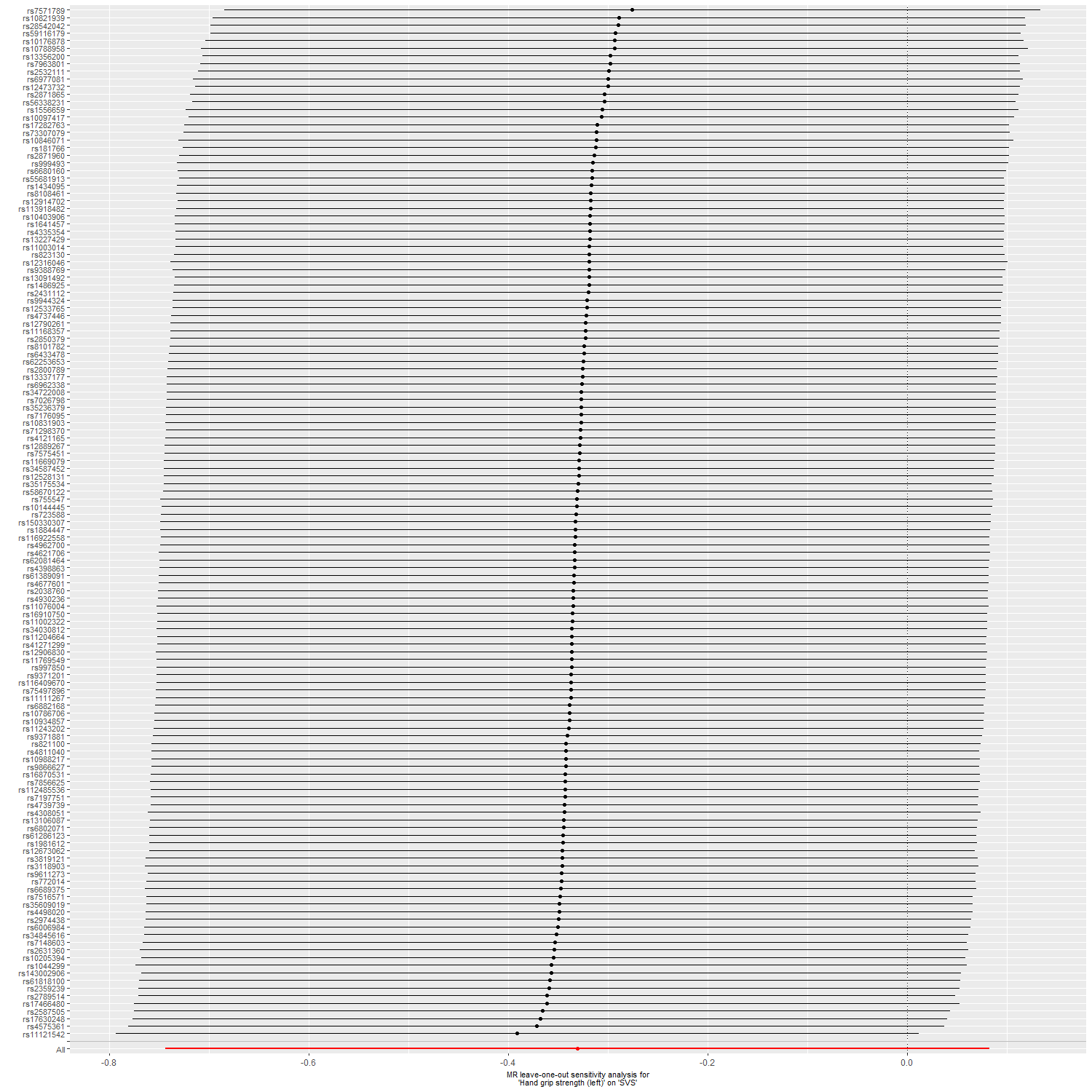


Supplementary Figure 21: Hand grip strength (left) on SVS.
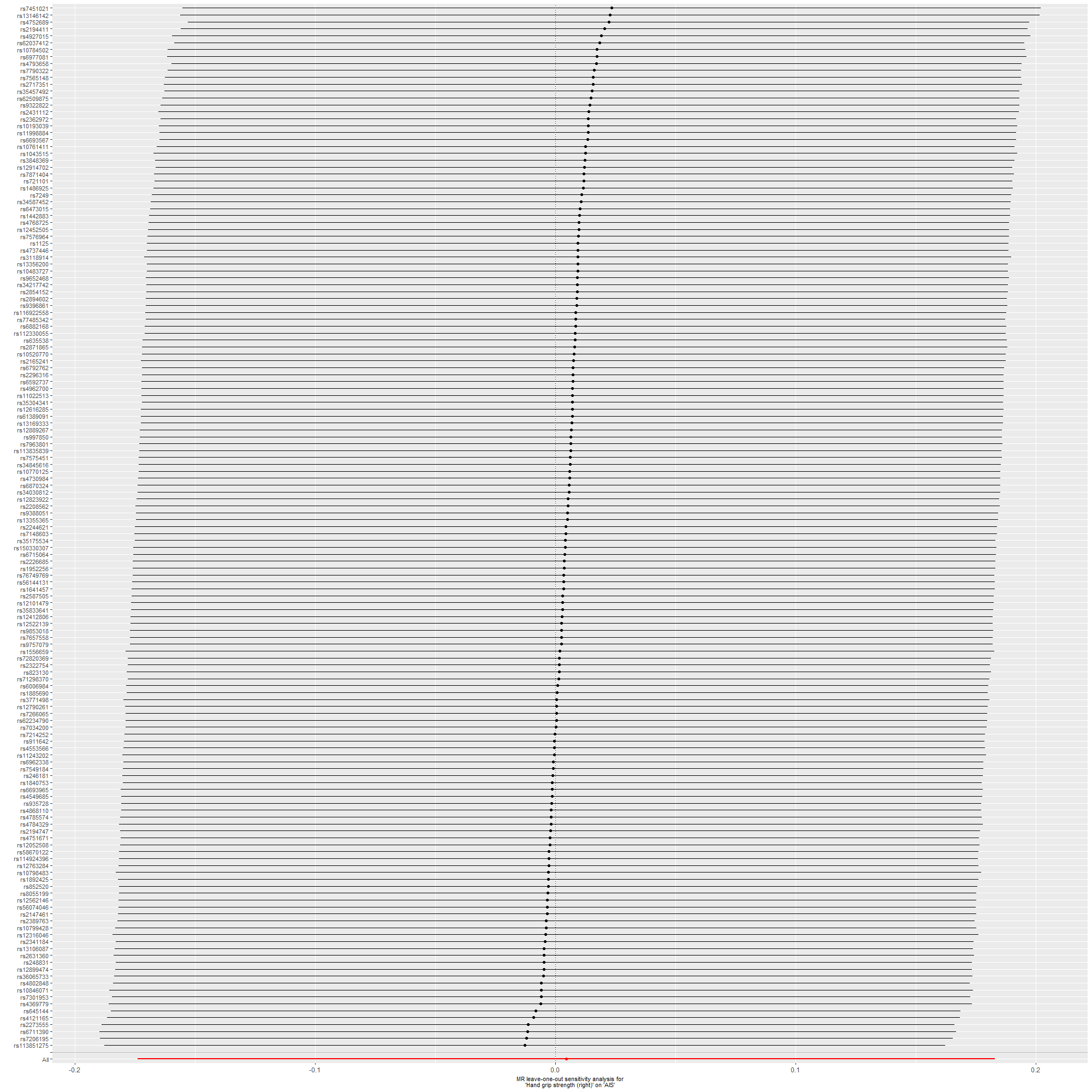


Supplementary Figure 22: Hand grip strength (right) on AIS.
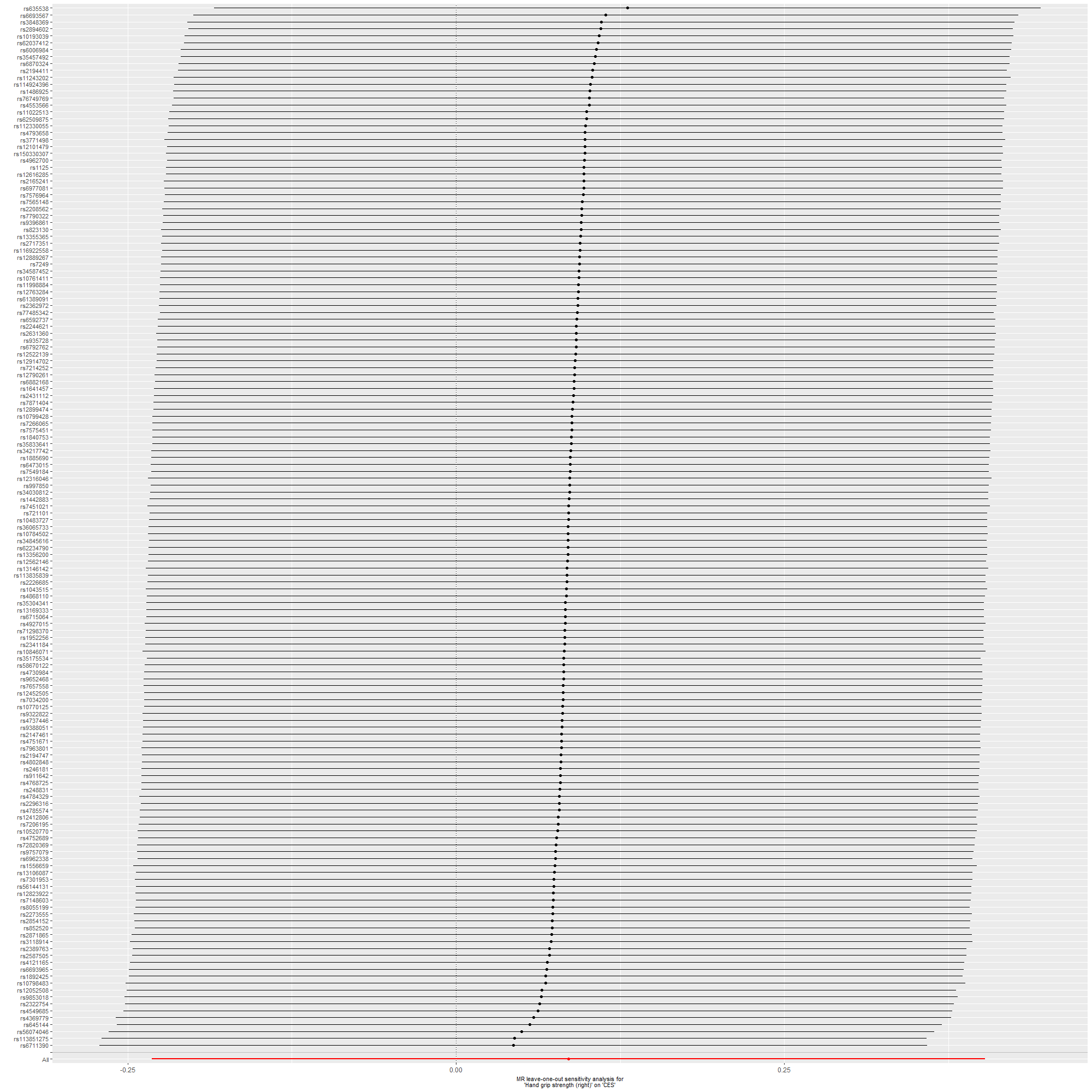


Supplementary Figure 23: Hand grip strength (right) on CES.
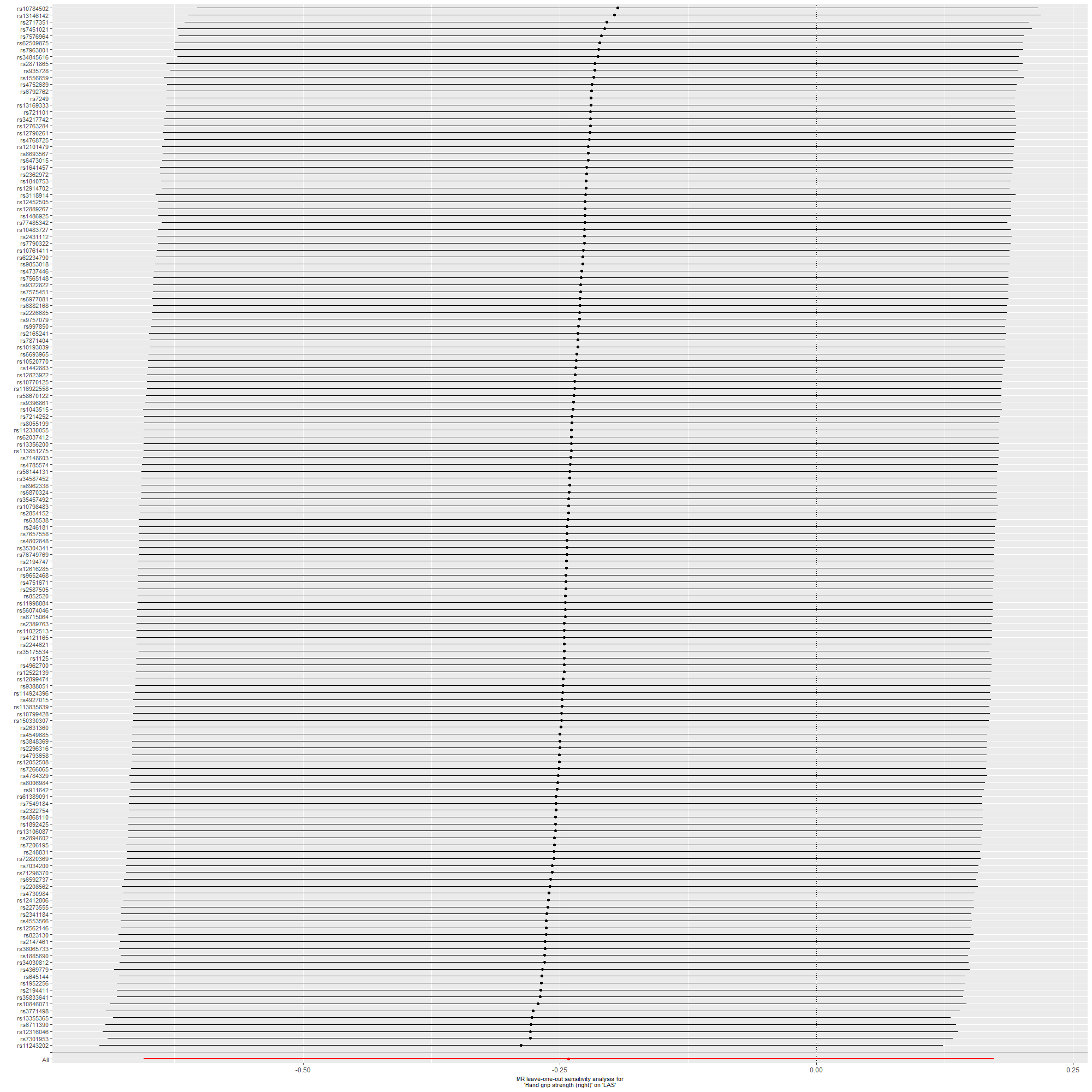


Supplementary Figure 24: Hand grip strength (right) on LAS.
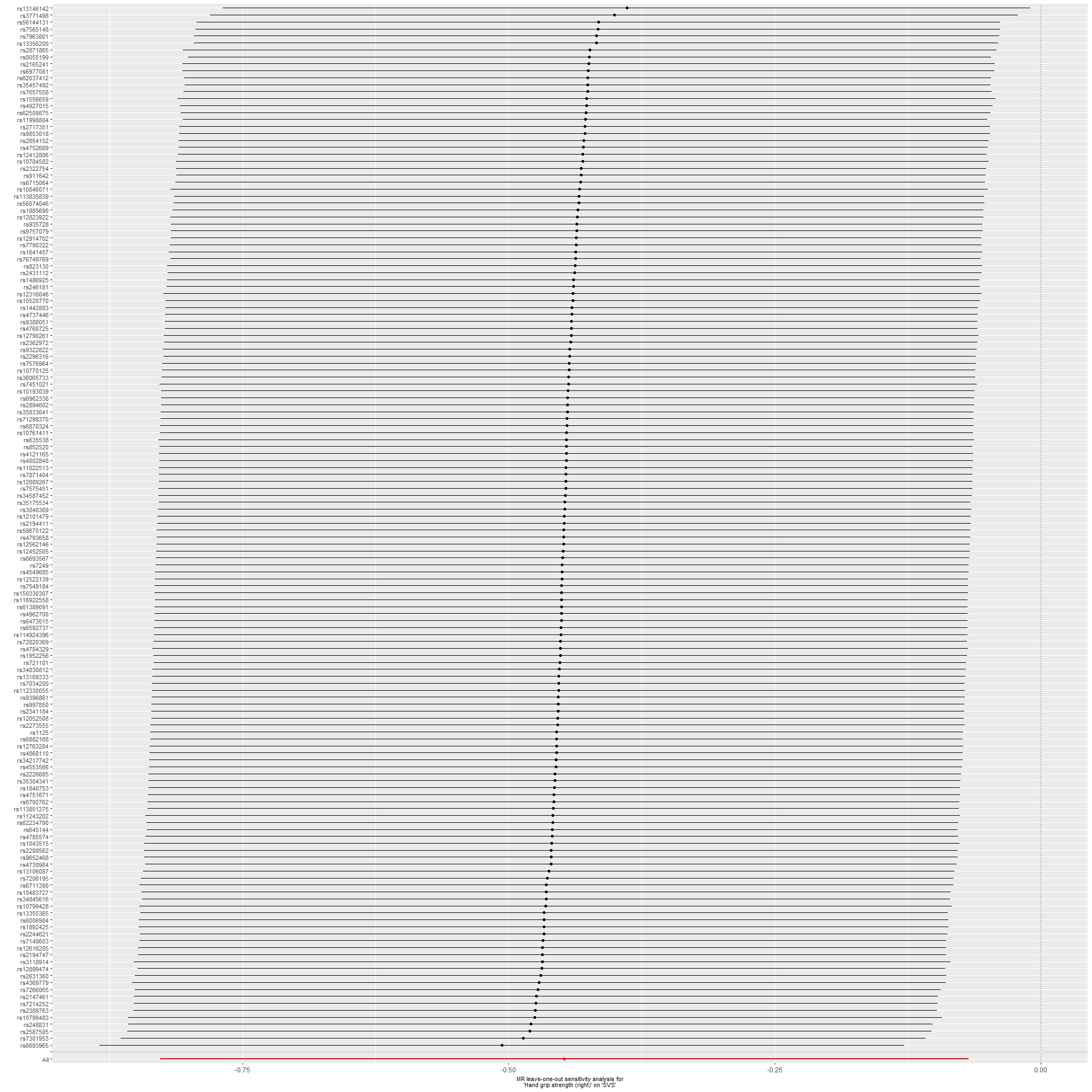


Supplementary Figure 25: Hand grip strength (right) on SVS.
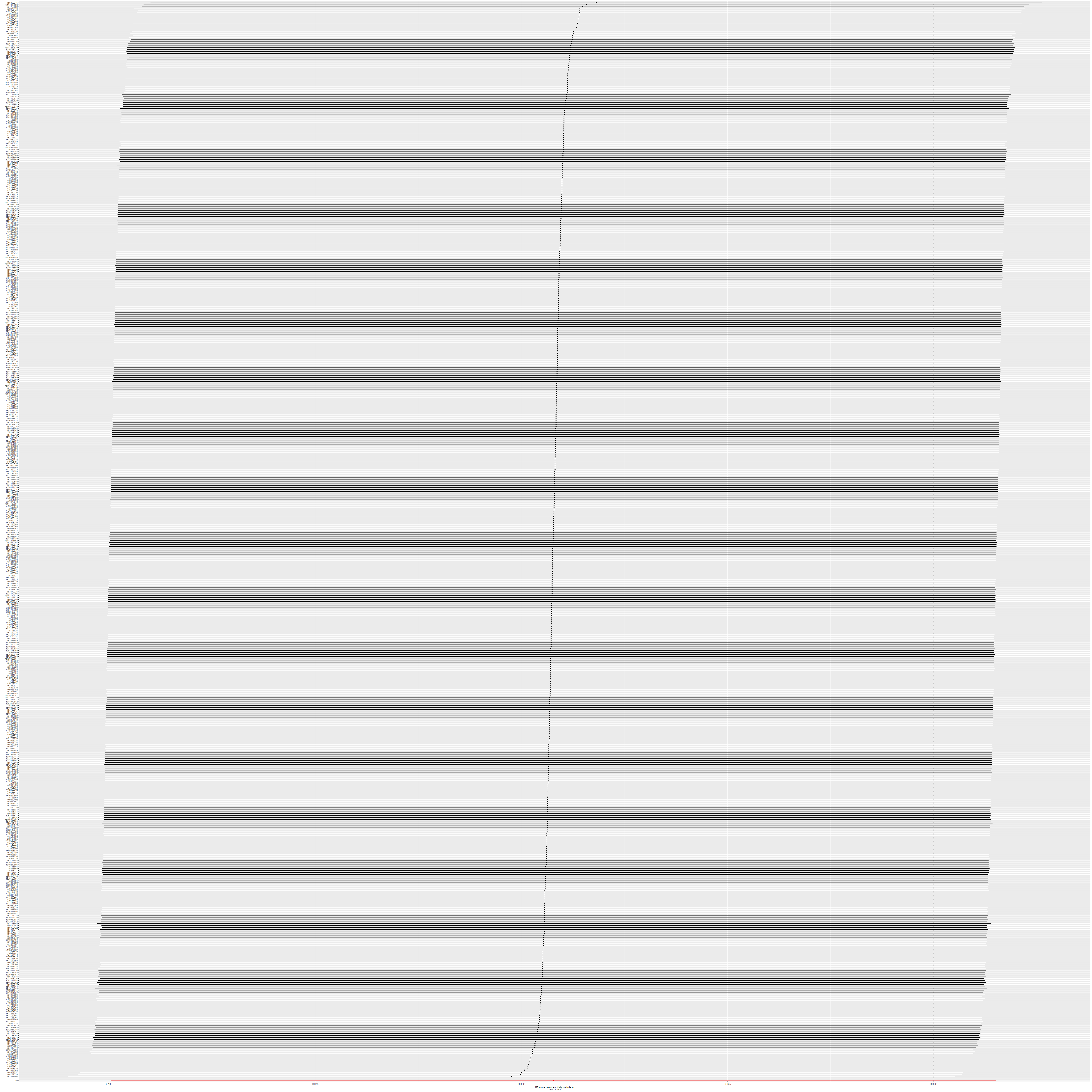


Supplementary Figure 26: ALM on AIS.
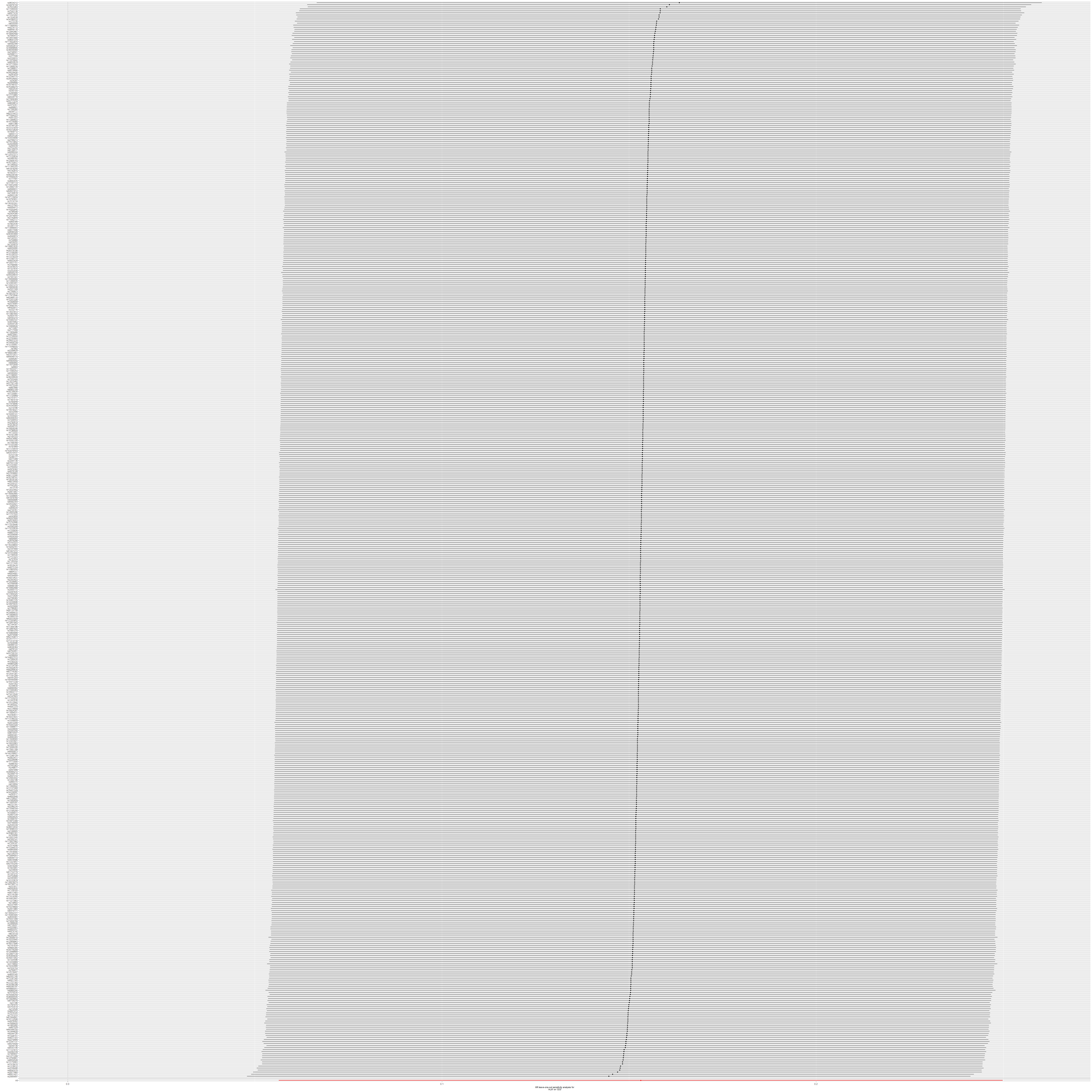


Supplementary Figure 27: ALM on CES.
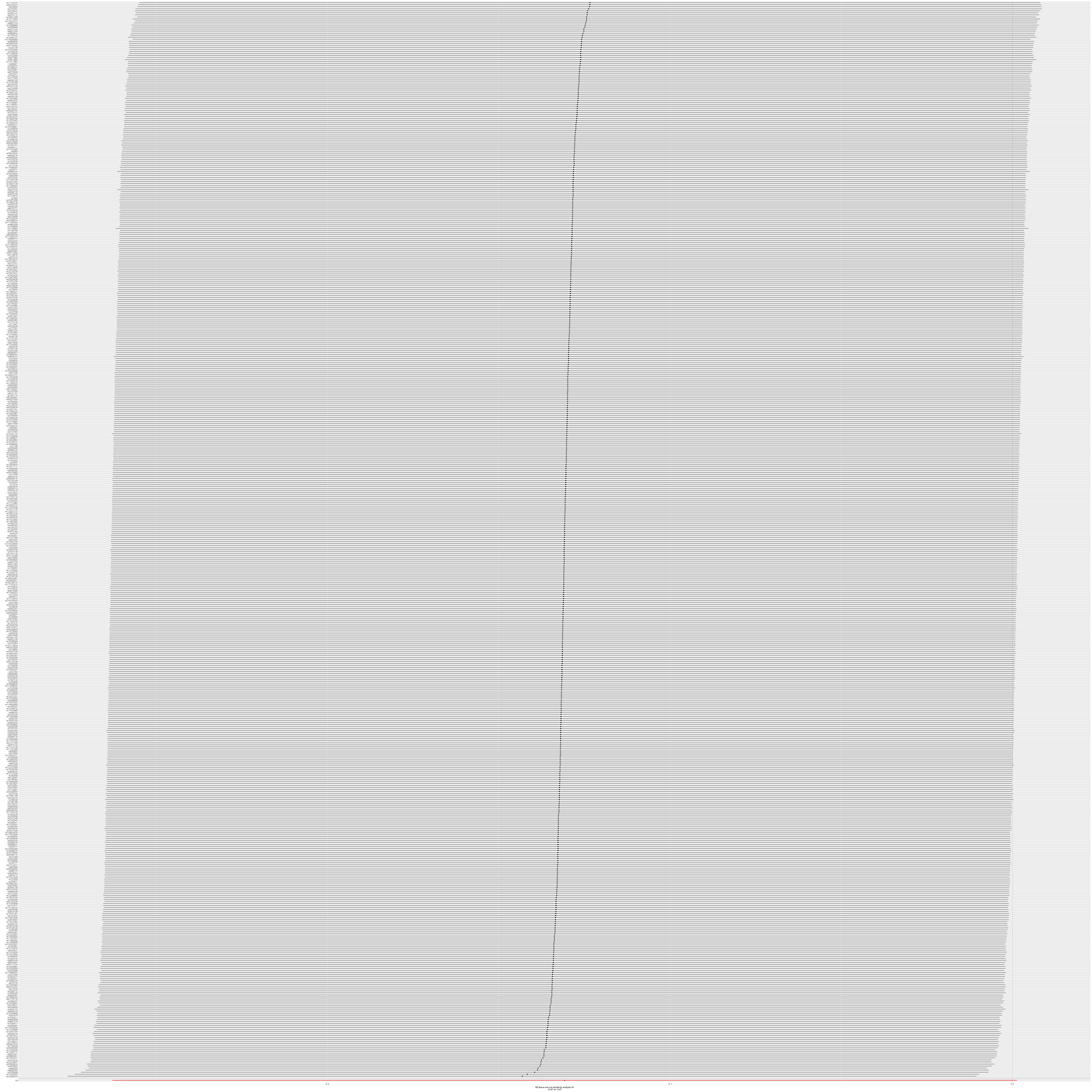


Supplementary Figure 28: ALM on LAS.
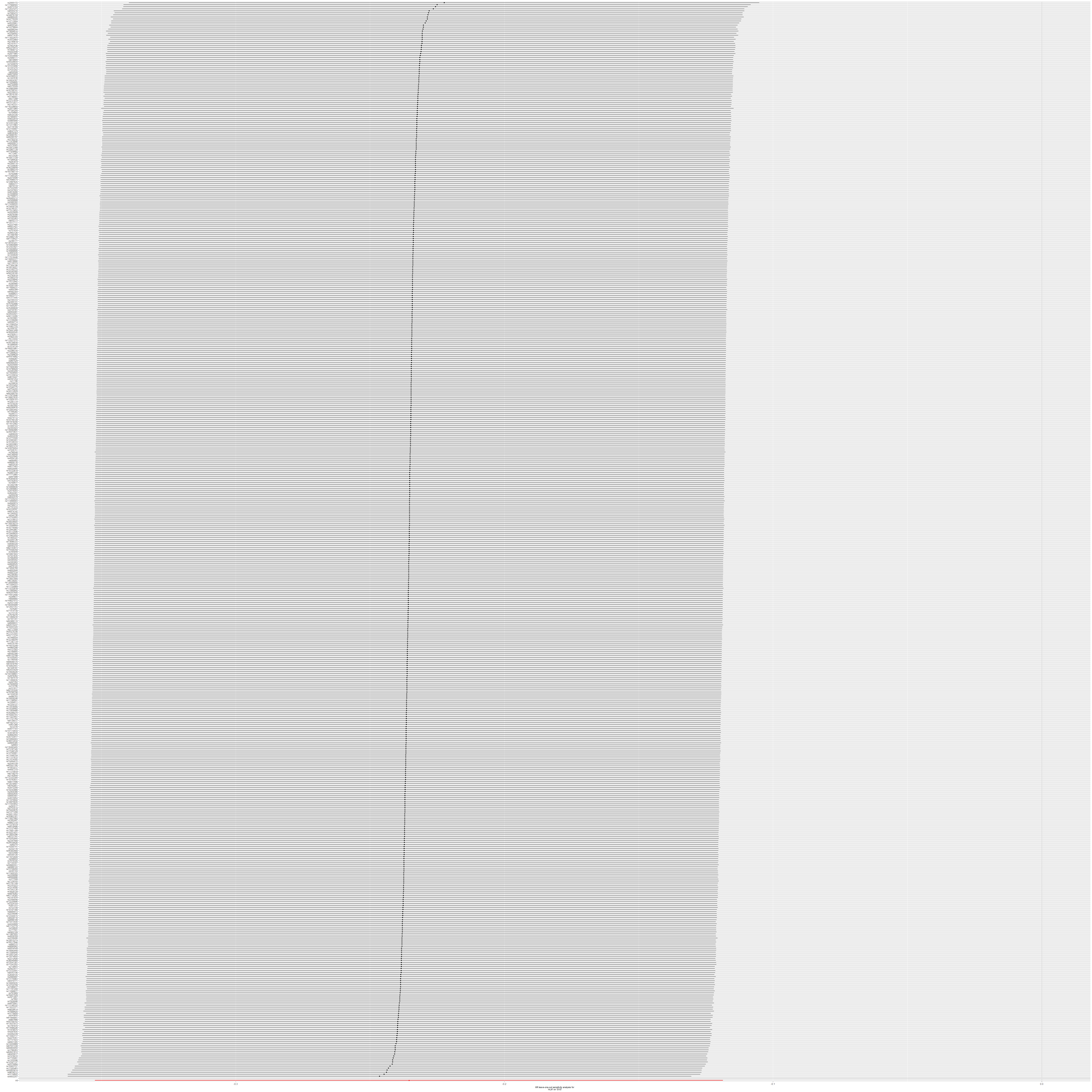


Supplementary Figure 29: ALM on SVS.
